# Differential response of finger millet accessions to contrasting saline water levels and irrigation regimes under desert conditions

**DOI:** 10.3389/fpls.2026.1754820

**Published:** 2026-02-27

**Authors:** Abidemi Talabi, Nhamo Nhamo, Sumitha Thushar, Prashant Vikram, Hifzurrahman Rahman, Mohammed Shahid, Neeru Sood, Malavika Sudheer, Dheeraj Thikkamaneni, Amna Almarri, Fatma Alsaffar, Deep Galani, Moyeez Alam, Sonia Goel, Rakesh K. Singh

**Affiliations:** 1 International Center for Biosaline Agriculture, Dubai, United Arab Emirates; 2 Innovation Oasis, Silal Food and Technology LLC., Abu Dhabi, United Arab Emirates; 3 ICAR - Indian Agricultural Research Institute, New Delhi, India; 4 Department of Biotechnology, Birla Institute of Technology & Science Pilani, Dubai, United Arab Emirates; 5 Zayed University, Dubai, United Arab Emirates; 6 Junagadh Agricultural University, Junagadh, Gujarat, India; 7 Olobion Omics Labs, Abu Dhabi, United Arab Emirates

**Keywords:** arid and semi-arid regions, drought stress, genetic stability, marginal environments, multi-traits genotype-ideotype distance index, salinity stress, stress tolerance index, water scarcity

## Abstract

Water salinity and scarcity constitute major limitations to crop production in arid and semi-arid regions. Introduction of nutritious and stress-tolerant underutilized crops is a promising approach for dietary enrichment, cropping system diversification, remediation of marginal and degraded lands, and building climate resilience. The primary objectives of this study were to investigate the effect of water salinity and managed water-deficit stress on grain and fodder yield, identify multi-trait ideotypes, and validate the stability and genetic gain in finger millet ideotypes over a 2-year period. A total of 80 finger millet accessions were evaluated under fresh water (0 dS/m) and two saline irrigation water (6 and 10 dS/m) in Dubai during the 2020/2021 cropping season. Validation of a selected elite subset was conducted under a combination of optimum, salinity, and drought-stress regimes (0 dS/m, 6 dS/m, 10 dS/m, and 50% irrigation) during the 2021/2022 cropping season. Initial analysis showed a grain yield (GYLD) reduction of 87% under 10 dS/m saline irrigation water compared with the control, and the genotype-by-treatment (G × T) interaction revealed highly significant effects for GYLD. Using multi-trait genotype–ideotype distance index (MGIDI), 20 elite accessions were identified, demonstrating a remarkable increase in mean GYLD under high saline irrigation water, corresponding to a genetic gain of 167% over the reference population mean. Validation trials confirmed the success of the selection by showing a non-significant G × T for GYLD and dry fodder yield (DFYLD) across the four validation treatments, alongside a significant increase in heritability (*H^2^*) for GYLD from 0.60 to 0.78. Comparative analysis revealed that managed water-deficit stress was the most limiting factor for GYLD in the elite subset, causing an average loss of 42.7% compared to 20.4% under high saline water irrigation. However, DFYLD displayed exceptional stability across both saline water and water-deficit stress types. The comparative analysis presented in Venn diagrams ultimately identified a core group of stable, broadly adapted accessions, including IE 4028 and IE 4570, which are recommended as high- impact parental lines for combined stress tolerance. These findings establish a reliable selection framework for enhancing the climate-resilience of underutilized crops in marginal environments.

## Introduction

Abiotic stresses associated with climate change, including drought, salinity, flood, heat, and nutrient deficiency, are increasingly affecting crop productivity globally. Rising temperatures, altered precipitation patterns, and increasing evapotranspiration are intensifying the frequency and severity of these stress factors. At the cellular and whole-plant levels, abiotic stresses disrupt metabolic homeostasis, primarily through osmotic stress, ionic toxicity, and the overproduction of reactive oxygen species (ROS) (Iqbal et al., 2025). Plant survival and adaptation under such conditions depend on tightly regulated stress signaling networks, including redox regulation, antioxidant defense systems, and hormone-mediated signaling pathways that coordinate growth–stress trade-offs. Recent review studies have highlighted the central role of redox signaling in integrating multiple abiotic stress responses and modulating tolerance mechanisms across diverse crop species (Iqbal et al., 2023, 2025; Iqbal and Yaning, 2024). Understanding these interconnected stress–response processes is therefore critical for developing climate-resilient crops capable of sustaining productivity under increasingly hostile environments.

Among the stresses mentioned earlier, soil salinity and water scarcity/drought are major contributors to the decline in agricultural productivity in arid and semi-arid regions, where these challenges directly threaten food and fodder production (Adamu et al., 2024). Salinity adversely affects several geographical locations globally. According to Mukhopadhyay et al. (2021), soil salinity had affected 33% of irrigated agricultural lands and 20% of land under cultivation globally with anticipated intensified reach by 2025. Salinity driven land and water degradation is bound to affect nearly 1 billion people, the majority of whom reside along coastal zones. In locations where seawater intrusion will intensify, food insecurity will increase, freshwater scarcity will deepen, ecosystem functions will be disrupted and livelihood opportunities destroyed (Wu et al., 2020; Tran et al., 2021). Furthermore, Park et al. (2018) projected that salinization could worsen under current climate change scenarios, with models predicting that salt-affected areas may expand to between 24% and 32% of the total land surface by the end of this century. The physiological and anatomical mechanisms of salinity tolerance in plants rely on the efficacy of the plants in salt transportation, uptake, and accumulation in tissues (Shabala and Munns, 2017). Plants use tolerance, resistance, and avoidance as the main mechanisms to prevent the accumulation of toxic Na+ concentrations in the leaves and to regulate concentrations of Na^+^, K^+^, and Cl^-^ within the various cell compartments (Franco-Navarro et al., 2025). Drought stress remains a critical challenge in arid and semiarid regions, significantly impacting crop productivity by restricting water availability and leading to reduced growth and yield (Shi et al., 2022). Crops under drought conditions experience alterations in physiological and biochemical processes, resulting in notable yield losses (Seleiman et al., 2021). While mitigation strategies for effective management of water resources may offer some relief, adaptation through the introduction of improved, nutritious, underutilized crops that can effectively withstand biotic and abiotic stresses represents an environmentally acceptable and economically viable approach to salvaging degrading agricultural lands (Gupta et al., 2017). This will ensure that food security concerns are addressed and also contribute to dietary diversification and the building of long-term resilience in vulnerable agricultural systems. Additionally, the global population growth predicted to exceed 9 billion people by 2050 demands an increase in food production by about 60% in order to support food security for all (Adam, 2021). Climate change and related land degradation (including seawater intrusion from rising sea levels) threaten to derail the targeted food production targets. Multiple crop stresses reduce the production potential as well as the quality of food (Raza et al., 2025).

In recent years, research aimed at introducing, testing, and identifying highly nutritious and stress-tolerant underutilized crops for dietary enrichment and diversification. Phytoremediation of marginal lands characterized by salinity, water scarcity, and heat stress has attracted attention as well. Among the underutilized crops being evaluated in line with the Food and Agriculture Organization of the United Nations Regional Office for Asia and the Pacific (FAO/RAP), small millets have emerged as particularly promising candidates due to their high nutritional profile, climate resilience, local availability, and economic viability (Li and Siddique, 2018). Finger millet (*Eleusine coracana* (L.) Gaertn.) stands out in this category due to its exceptional nutritional composition and remarkable tolerance to multiple abiotic stresses. Finger millet is characterized by high calcium content (0.34%), substantial dietary fiber (18%), protein (6%–13%), and phenolic compounds (0.3%–3%), making it a valuable nutri-cereal for addressing malnutrition (Chandra et al., 2016). Furthermore, finger millet possesses a C4 photosynthetic pathway that enables efficient water and nitrogen utilization under hot and arid conditions without severely affecting yield (Hittalmani et al., 2017). A particularly valuable characteristic of finger millet is its dual-purpose nature, providing both grain for human consumption and high-quality fodder for livestock feeding (Gupta et al., 2017). These attributes, combined with its drought and salt tolerance superiority compared to many staple cereals, make finger millet a priority crop for stress breeding programs. However, while this crop is known for its resilience, it is surprising that limited data is available on how it performs under the extreme saline water irrigation in hyper-arid desert conditions. It is often labeled as “stress-tolerant” in a general use, but the actual limits of this tolerance and the differences in responses of genotypes need to be quantified/validated. This study aims to move beyond these generalities to identify specific genotypes of finger millet that can deliver substantial harvest where traditional staples would fail, particularly focusing on the balance between grain and fodder production which is critical for local farmers.

Effective germplasm characterization and classification form the foundation of successful breeding programs. Plant breeders routinely classify accessions into clusters based on similarities in trait expression, which allows for strategic selection either within homogenous clusters or across clusters to ensure genetic diversity (Dwivedi et al., 2017). However, the effectiveness of this approach depends critically on accurate characterization of germplasm under target stress conditions. For finger millet, while genetic diversity has been well documented, the functional grouping of accessions based on their performance under salinity and water-deficit conditions remains poorly understood. Understanding how different accessions respond to varying levels of saline water and drought stress is essential for identifying stable and broadly adapted genotypes suitable for cultivation in marginal environments.

It is noteworthy that plants have different mechanisms for coping with abiotic stress inclusive of drought and salinity. For example, drought and salinity have been reported to induce oxidative stress in wheat; thus, to select varieties with stress tolerance, focus is on lines with enhanced cell membrane stability and higher yields under stress conditions (Naqi et al., 2025). According to Bijalwan et al. (2021), watermelon employs a combination of mechanisms such as stomata regulation and tolerance to salinity and high antioxidant enzyme levels to cope with exposures to abiotic stresses.

The complexity of breeding for grain yield under stress conditions arises from its polygenic nature and typically low heritability, which render direct selection for yield alone ineffective (Yadav et al., 2010; Fischer and Rebetzke, 2018). Consequently, breeders seek to identify important secondary traits that are genetically correlated with grain yield and can serve as indirect selection criteria for yield improvement under stress conditions (Talabi et al., 2017). Alternatively, important secondary traits can be combined with grain yield through multi-trait selection indices for ideotype design (Olivoto and Nardino, 2021). This approach recognizes that trait interrelationships depend on both the genetic materials involved and the environmental conditions under which evaluations are conducted. Furthermore, genotype-by-treatment interactions are frequently significant across different stress levels, necessitating the identification of stress-specific secondary traits for effective selection (van Eeuwijk et al., 2016). Some accessions exhibit specific adaptation to particular saline water levels, while others demonstrate broader adaptation across a range of saline water levels and drought intensities.

Given the importance of rigorous germplasm screening to identify robust sources of salt tolerance and effective selection strategies, we hypothesize that genotype-by-treatment interactions will be significant across different saline water levels and water-deficit stress, necessitating the identification of stress-specific secondary traits and the use of multi-trait selection indices to effectively select stable and superior finger millet accessions. The objectives of the present study were to (i) identify superior finger millet accessions with high grain and/or fodder yield under varying saline water levels and water-deficit stress and quantify stress-induced yield reduction, (ii) investigate the interrelationships among traits across contrasting research conditions, and (iii) assess the stability of accessions for grain and/or fodder yield across stress and optimum research conditions to recommend climate-resilient varieties for registration and commercialization.

## Materials and methods

### Genetic material

A total of 80 traditional cultivars/landraces of finger millet, received from the International Crops Research Institute for the Semi-Arid Tropics, India, were used for this study.

### Initial screening and field evaluation

The initial screening utilized 75 unreplicated test entries and five replicated check entries, evaluated using an augmented randomized complete block design (A-RCBD). The experimental layout consisted of five blocks per environment. The five check entries were replicated once in each of the five blocks (total of 25 check plots, i.e., five check entries × five replications), while the 75 test entries were randomly assigned to the remaining unreplicated plots within the blocks. This entire A-RCBD structure was deployed independently under three contrasting saline irrigation water treatments [0 dS/m (control), 6 dS/m, and 10 dS/m], resulting in a total of 300 experimental plots (100 plots/environment × three environments). Each experimental unit measured 1 m^2^, with inter-row and within-row spacing of 0.25 m. This experiment was conducted at the research field of ICBA (25.0947° N, 55.3899° E; elevation, 30 m asl) from January to May, 2021. Climatic data on the temperature, relative humidity, and rainfall at the study location during the trial periods are detailed in **Supplementary Table S1**.

The experimental soil was sandy, and animal manure was applied at 3 kg/m^2^ prior to planting. Drip irrigation pipes supplied at the rate of 50–80 m^3^/ha daily at the respective salinity levels, which were set and monitored through the Supervisory Control and Data Acquisition (SCADA) system. Seedlings were thinned to one per hill at 3 weeks after planting (WAP), resulting in a final population density of 166,000 plants per hectare. Compound fertilizer (NPK: 15–15–15) was applied at 30 kg/ha for N, P, and K at 4 WAP, with an additional 30 kg/ha of N applied for top-dressing at 7 WAP. Weeds were manually controlled, and insect pests were managed using the systemic pesticide Confidor (imidacloprid, 200 g/L at 5 l/ha) as applicable. **Supplementary Table S2** presents information on the soil properties of the experimental field before conducting the experiments.

### Validation trial for an elite subset of finger millet genotypes

From the 80 genotypes, 20 elites were selected based on their superior performance in the initial screening under saline irrigation water stress. These 20 lines were subsequently evaluated under four independent treatment conditions (0 dS/m at 100% irrigation—control, 6 dS/m at 100% irrigation, 10 dS/m at 100% irrigation, and induced water-deficit stress—50% irrigation at 0 dS/m). The validation trial was organized using a randomized complete block design (RCBD). The experimental unit and all crop husbandry practices remained the same as the previous experiment. For 50% irrigation, the plants received the same amount of water as the control until 14 days after planting, when the induced water-deficit stress was imposed so that plots under this stress received only half the quantity of water applied to the control i.e., at 100% irrigation, and this was sustained until the end of the crop cycle. This design was intentionally structured to distinguish the individual effects of salinity from those of water deficit. By testing these stresses independently under the same environmental conditions, we were able to compare the specific yield penalties caused by high salinity (osmotic/ionic stress) with those caused by induced water stress. The comprehensive chemical characterization of irrigation water used in ICBA field experiments (Dubai, UAE) for year 2023/2024 season is presented in **Supplementary Table S3**. Irrigation water samples were analyzed for pH, electrical conductivity (ECw), total dissolved solids (TDS), major cations (Ca²^+^, Mg²^+^, Na^+^, and K^+^), and major anions (Cl^-^, SO_4_²^-^, HCO_3_^-^, and CO_3_²^-^) in accordance with *Standard Methods for the Examination of Water and Wastewater* (24th ed.) (Lipps et al., 2023). Sodium adsorption ratio (SAR) and residual sodium carbonate (RSC) were calculated from measured ionic concentrations following FAO irrigation water quality guidelines (FAO, 2020). All analyses were conducted at the Central Analytical Laboratory of ICBA, Dubai, UAE. The actual chemical compositions were determined for 0.6, 5, and 15 dS/m. However, the compositions for 6 and 10 dS/m were derived based on known compositions at 0.6, 5, and 15 dS/m by interpolation approach. A linear relationship was assumed between salinity and ionic concentration since lower salinity levels are attained by diluting ground water of high salinity of about 20 dS/m with fresh water (0.6 dS/m) to attain the desired lower salinity levels.

### Data collection

Data was collected on plant height (PHT) as the average length of five sample plants from the base of the plant to the tip of the finger. Plant aspect was a visual score on the acceptability of the cultivar on a scale of 1 to 9. This was based on the overall plant appeal including plant type/architecture, uniformity of plant heights, uniformity of plant and finger color/type, productiveness of fingers, freedom from disease and insect damage, and lodging resistance (1 = excellent, 9 = completely undesirable). Days to flowering (FLW) was measured as the number of days after planting when 50% of the plants in each plot have fingers that were shedding pollen. Days to maturity (DM) was measured as the number of days after planting when 50% of the plants in the plot were ready for harvest. Harvesting was done only from plants within 0.75 m^2^ such that border rows were excluded. Stand count (SC) was recorded as number of plants within the net plot area 2 weeks before harvesting. Lodging was scored based on the percentage of plants within plot tilting more than 30° from the vertical on a scale of 1 to 10, where 1 = 10% of plants are lodged and 10 = all plants are lodged. All of the traits involving visual assessment scores were performed independently by three experienced scientists for each plot. To ensure the reliability of the data, the final score recorded for analysis was the consensus value reached by all three evaluators after cross-validation of their individual assessments. This multi-rating approach was employed specifically to minimize individual bias and improve the accuracy of the qualitative scoring. Panicle weight (PWT) was measured as the weight of the panicles in kilogram per harvest area of 0.75 m^2^, converted to kilogram per hectare. Fresh and dry fodder yields were the recorded as the weight of the fresh and dry fodders in kilogram per harvest area, converted to kilogram per hectare. The percentage of grain moisture at harvest was determined using OGA digital grain moisture meter. Grain yield (GYLD) was measured as the weight of the threshed kernels per harvest area in tons, which was converted to tons per hectare, and 12.5% moisture content was determined using Equation 1 below:

(1) $$Grain\;yield\;\left(\frac{t}{ha}\right)=\frac{Grain\;yield\;per\;plot\;\left(t\right)\;\times \left(100-87.5\right)}{Moisture\;content\;\times Plot\;area}$$

### Statistical analysis of data

#### Data quality control and model fitting for the screening trial

All analyses were performed using R version 4.4.2 (Team, R. C, 2023) within the RStudio environment, employing the packages lme4 (Bates et al., 2015), tidyverse (Wickham et al., 2019), metan (Olivoto and Lúcio, 2020), and ggplot2 (Gómez-Rubio, 2017).

Data from the initial screening experiment conducted under three saline water irrigation levels (0, 6, and 10 dS/m) were first subjected to rigorous quality control. The dataset, based on the augmented randomized complete block design (A-RCBD), contained replicated check entries and unreplicated test entries within each salinity level. To account for local environmental heterogeneity, an adjusted RCBD analysis was implemented within each environment to obtain adjusted means (BLUEs) for all test entries. These adjusted means were subsequently used as the input for stress tolerance index (STI) and MGIDI calculations.

#### Analysis of variance using the combined linear mixed-effects model

To assess the performance of all finger millet genotypes across saline water levels and partition the sources of variation, the raw-plot-level data was used to fit a linear mixed-effects model (LMER) using the lme4 package. The model was defined as shown in Equation 2.

(2) $$Y_{ijk}\;=u+T_{i}+\;B_{j}\left(T_{i}\right)+\;G_{k}+{\left(G\;x\;T\right)}_{\;i\left(k\right)}+\;E_{ijk}$$

where

*Y_ijkn_* = observed phenotypic value for trait; *T_i_* = fixed effect of the *i*-th salinity level; *B_j_*(*T_i_*) = random effect of the *j*-th block nested within *i*-th treatment; *G_k_* = random effect of the *k*-th genotype; (*G* × *T*)*_ik_* = random genotype-by-treatment interaction; *E_ikjn_* = residual experimental error.

The model served two primary purposes. The first being that the significance of the fixed treatment effect (salinity) was assessed via *F*-tests using ANOVA with the Satterthwaite approximation to calculate the effective degree of freedom. The second was that the significance of the random terms (genotype and genotype-by-treatment interaction—*G* × *T*) was assessed via likelihood ratio tests (LRT) comparing nested models, and variance components were extracted.

#### Estimation of genetic values and stress tolerance index

For each trait, best linear unbiased predictors (BLUPs) were extracted from the full Combined LMER model (fitted to raw data) for each genotype × treatment combination. These BLUPs, combined with the fixed-effect means, provided the predicted genetic value (BLUE) adjusted for all design effects (block and environmental heterogeneity). These final genetic values were used for downstream analyses. Negative predicted values from any trait were truncated to zero to preserve biological relevance. The stress tolerance index (STI) was then computed for grain yield to identify and select genotypes that are potentially stress tolerant for ideotyping using Equation 3.

(3) $$Stress\;Tolerance\;index\;\left(STI\right)=\frac{grain\;yield\;\left(control\right)\;x\;grain\;yield\;\left(severe\;stress\right)}{{\left(mean\;grain\;yield\;under\;control\;\right)}^{2}}\;$$

### Multi-trait ideotyping using multi-traits’ genotype–ideotype index

Before proceeding to ideotyping—to select the final genotypes that combine high yield potential with agronomic characteristics under high saline water concentration (10 dS/m), we conducted principal component analysis (PCA) to identify the traits that influence the total variation the most. These traits were used for the final selection of the salt-tolerant genotypes for validation. To achieve this, the multi-trait genotype–ideotype distance index (MGIDI) (Olivoto and Nardino, 2021) was computed using the Metan package. The data containing the BLUEs of the selected traits were first rescaled between 0 and 100 to align all traits in the same direction of the selection (higher values = desirable performance). Correlated traits were then reduced into orthogonal latent factors using factor analysis with varimax rotation, retaining components with eigenvalues greater than one. We finally calculated the MGIDI using Equation 4.

(4) $$MGIDI=\;\sum_{i=1}^{f}{\left[{\left(Y_{ij}-Y_{Ij}\right)}^{2}\right]}^{0.5}$$

where MGIDI is the multi-trait genotype–ideotype distance index for the *i*-th genotype; *Y*_ij_ is the score of the *i*-th genotype in the *j*-th factor; and *Y*_Ij_ is the score of the ideal genotype (ideotype) with maximum rescaled values (100) across all traits. Genotypes with lower MGIDI values were considered closer to the ideotype.

A selection intensity of 35% was applied to identify elite genotypes combining high grain and fodder yield, early maturity, and desirable plant architecture under saline conditions.

#### Validation trial analysis

The 20 genotypes selected from the screening stage were re-evaluated under four conditions (0, 6, and 10 dS/m and 50% irrigation) using a randomized complete block design (RCBD). Data analysis aimed to confirm genotype performance and identify trait patterns across salinity and water-deficit stresses.

#### Combined linear mixed model for validation and principal component analysis

A combined linear mixed model (LMM) was fitted to assess the significance of genotype, treatment, and their interaction effects using Equation 5.

(5) $$Y_{ijk}\;=u+T_{i}+\;B_{j}\left(T_{i}\right)+\;G_{k}+{\left(G\;x\;T\right)}_{\;i\left(k\right)}+\;E_{ijk}$$

where the terms are as previously defined. However, treatment and genotype effects were modeled as fixed, while block was random, consistent with the confirmatory objective. The significance of the *G* × *T* term was used to infer treatment-specific adaptation.

In this model, genotype (G), treatment (T) and their interaction were made fixed effects, while block nested within treatment was made a random effect. The significance of these fixed effects was evaluated using Wald *F*-tests. In addition to the combined model, single or treatment-based model was also fitted using similar model parameters, though without interaction effect. In each of the single model, genotypes were also made a fixed effect, while block was random. From the combined and single mixed models, we extracted the combined and treatment-based BLUEs. The overall BLUEs were standardized and subjected to principal component analysis (PCA) to identify the traits that are major contributors to the phenotypic variation for selection and inclusion in a further analysis. Traits with loadings ≥0.5 on the principal components with Eigen values ≥1 were retained for further analysis.

#### Genotype-by-trait analysis, correlation, and identification of consistent multi-trait superior genotypes

The selected traits from the PCA analysis was used for genotype-by-traits analysis using the function “fviz_pca_biplot” in the factoextra package (Kassambara and Mundt, 2020). From the BLUEs extracted from the single models (treatment-based), grain and dried fodder yields were visualized using boxplots. The statistical significance among the treatment in the boxplots was declared through nonparametric Kruskal–Wallis tests. To control type I error, the *p*-values from this test was corrected using false discovery rate (FDR) correction (Benjamini and Hochberg, 1995). Additionally, a correlation analysis was conducted to identify consistent multi-traits superior genotypes. The MGIDI index was re-applied on the single model BLUEs to identify superior genotypes under zero stress (control), high salinity (10 dS/m), and induced water-deficit stress (50% irrigation) conditions. From the selection results, overlapping genotypes across the three conditions were visualized using Venn diagrams generated by the ggVennDiagram package (Gao et al., 2021).

## Results

### Analysis of variance and mean performance from preliminary trial evaluation

The fixed effect part of the combined ANOVA model involving salinity treatment (**Table 1**) revealed that the imposed salt gradient significantly impacted the overall performance of the finger millet germplasm. The *F*-test for the treatment effect was highly significant (*p* < 0.001) for 10 out of the 11 traits measured, confirming the efficacy of the stress conditions in discriminating phenotypic responses.

**Table 1:** Descriptive statistics (mean and standard error) and treatment effect from analysis of variance of varying saline water levels on 80 finger millet accessions.

Trait	Control (0 dS/m)	Mild salinity (6 dS/m)	High salinity (10 dS/m)	Treatment effect	*P*-value	DDF
GYLD (t/ha)	2.19 ± 0.17	1.87 ± 0.17	0.29 ± 0.17	16.28	***	13.46
DFYLD (t/ha)	10.80 ± 0.46	9.55 ± 0.46	6.97 ± 0.46	279.75	***	10.84
FFYLD (t/ha)	35.80 ± 1.22	29.98 ± 1.22	16.31 ± 1.22	4,018.16	***	12.51
PHT (cm)	82.81 ± 2.19	74.2 ± 2.19	57.13 ± 2.19	3,010.50	***	11.18
DM	119.2 ± 0.85	118.96 ± 0.85	124.77 ± 0.85	729.55	***	10.98
PASP	4.77 ± 0.16	4.45 ± 0.16	5.13 ± 0.16	4.11	**	9.84
PANWT (t/ha)	3.55 ± 0.22	3.35 ± 0.22	0.83 ± 0.22	74.86	***	11.51
Lodging	3.77 ± 0.12	3.85 ± 0.12	3.53 ± 0.12	0.91	ns	13.40
FLW	94.80 ± 1.37	90.05 ± 1.37	93.55 ± 1.37	199.78	*	11.72
FH	181.29 ± 7.2	142.58 ± 7.2	142.58 ± 7.2	15.07	***	11.82
BH	181.29 ± 7.2	142.58 ± 7.2	142.58 ± 7.2	25,757.00	***	13.31

GYLD, grain yield; DFYLD, dried fodder yield; FFYLD, fresh fodder yield; PHT, plant height; DM, days to maturity; PASP, plant aspect; PANWT, panicle weight; FLW, days to flowering; FH, finger height; BH, bearing heads; ns, not significant (*P* > 0.05); *, significance at probability of 0.05; **, significance at probability of 0.01; ***, significance at probability of 001.

A sharp and progressive decline in the mean performance of most yield-related and vegetative traits was observed as the salinity increased from 0 to 10 ds/m. Grain yield (GYLD) was highly significantly affected (*p* < 0.001), reducing drastically from a mean of 2.19 t/ha under control conditions to only 0.29 t/ha under high salinity. Similarly, both dry fodder yield (DFYLD) and fresh fodder yield (FFYLD) exhibited highly significant decreases (*p* < 0.001), with FFYLD reducing by over 50% from the control mean of 35.80 t/ha. Vegetative growth was also constrained, as evidenced by the highly significant reduction (*p* < 0.001) in plant height (PHT), which decreased from 82.81 to 57.13 cm. The most reduction was seen in panicle weight (PANWT), which dropped by approximately 77% under high saline water stress conditions (*p* < 0.001).

Furthermore, a few traits displayed either stability or counter-intuitive response—for example, days to maturity (DM) was highly significantly affected (*p* < 0.001), showing a slight but significant increase from 119.2 days in the control to 124.77 days in the high salinity environment (stress delay in phenology). Plant aspect (PASP) was also significantly affected (*p* < 0.01), increasing slightly under high stress. Notably, lodging was the only trait that was not significantly affected by the salinity treatments, suggesting that the imposed stress levels did not substantially compromise the structural integrity of the stems across the population (**Table 1**).

The random part of the combined model (**Table 2**) revealed that significant genetic variation exists among the finger millet accessions for all traits, as indicated by the highly significant p(G) values (*p* < 0.05–0.001). More importantly, genotype-by-treatment interaction (G × T) was highly significant for GYLD (*p* < 0.001) and significant for fresh fodder yield (FFYLD), lodging, flowering (FLW), and bearing heads. This result confirms that the relative ranking of genotypes for these five key traits shifts significantly across the salinity levels. Conversely, the G × T interaction was non-significant for several other traits including DFYLD, PHT, DM, PASP, and PANWT. This confirms the stability of the genotypes in the expression of these traits across the saline water treatment levels.

**Table 2:** Variance components, likelihood ratio test (LRT), and broad-sense heritability estimates of finger millet traits across salinity treatments.

Source of variation	GYLD (t/ha)	DFYLD (t/ha)	FFYLD (t/ha)	PHT	DM	PASP	PANWT (t/ha)	Lodging	FLW	FH	BH
Variance components											
Genotype (G)	0.38	3.89	10.88	46.45	14.07	0.82	0.59	0.13	45.95	0.20	1,744.06
G × T	0.30	1.55	12.68	0.00	3.86	0.00	0.18	0.15	9.32	0.05	618.48
Block (T)	0.08	0.08	2.97	17.00	0.50	0.04	0.12	0.03	3.58	0.01	24.03
Residual	0.37	11.68	53.73	72.7	34.78	0.51	1.34	0.38	42.53	0.26	1,518.2
P-values (LRT)											
*P*(G)	***	***	*	***	***	***	***	**	***	***	***
*P*(G × T)	***	ns	*	ns	ns	ns	ns	**	**	ns	***
Genetic parameters											
Broad-sense heritability (*H*^2^)	0.60	0.32	0.29	0.34	0.34	0.60	0.34	0.40	0.55	0.49	0.61

GYLD, grain yield; DFYLD, dried fodder yield; FFYLD, fresh fodder yield; PHT, plant height; DM, days to maturity; PASP, plant aspect; PANWT, panicle weight; FLW, days to flowering; FH, finger height; BH, bearing heads; ns, not significant (*P* > 0.05); *, significance at probability of 0.05; **, significance at probability of 0.01; ***, significance at probability of 001.

Broad-sense heritability (*H*^2^) estimates ranged from a low of 0.29 for FFYLD to a high of 0.61 for BH. Some traits critical for selection, such as GYLD (0.60) and PASP (0.60), exhibited high heritability. This is a measure of the phenotype as a predictor of genetic value. Traits with moderate heritability include DFYLD (0.32), PHT (0.34), DM (0.34), PANWT (0.34), lodging (0.40), and FH (0.49). Moderate heritability suggests that while selection is still possible, the expression of these traits is influenced by environmental factors (**Table 2**).

### Salinity effect on grain and fodder yield of 80 finger millet accessions evaluated under varying saline water levels

**Figure 1:**
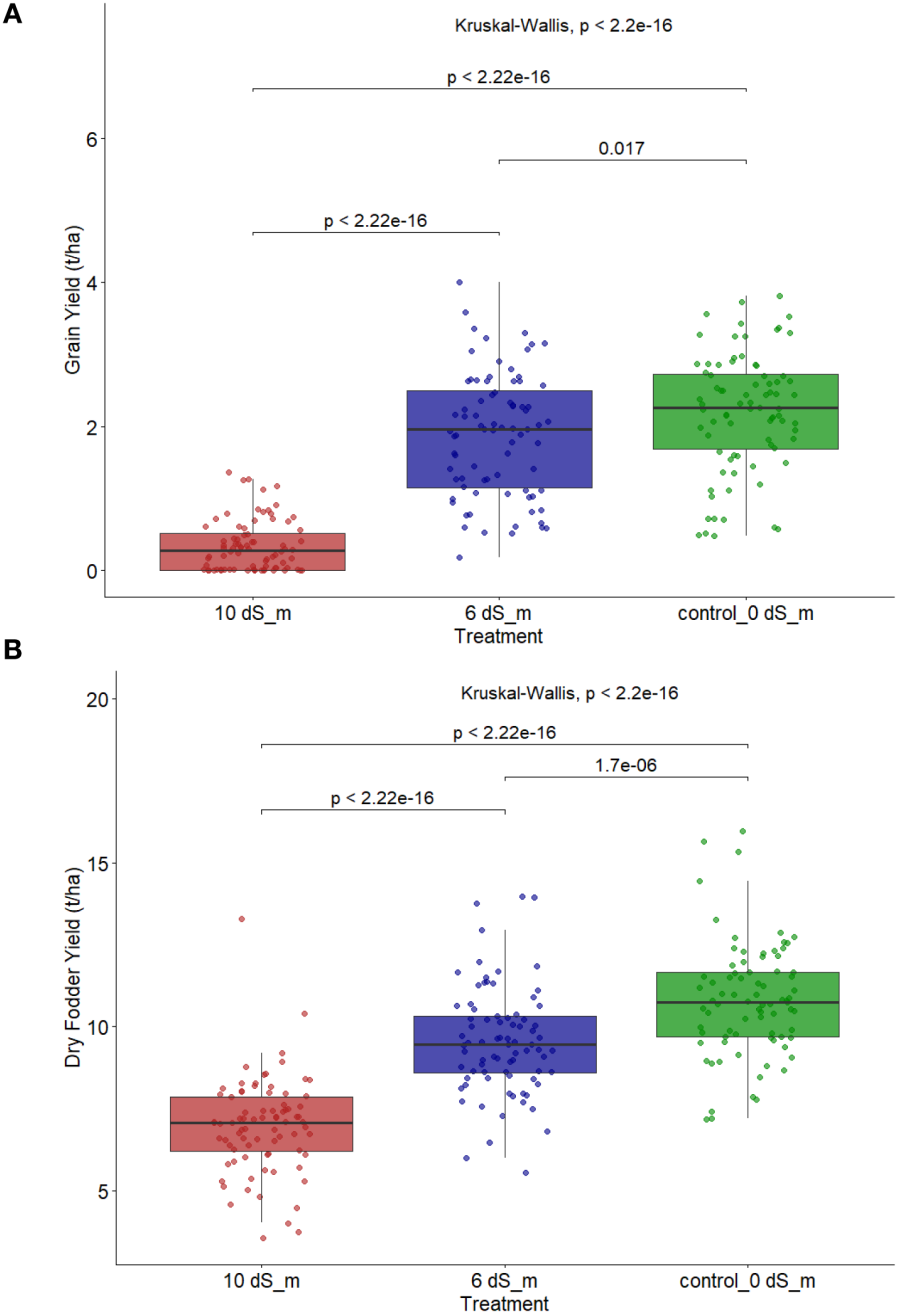
Box plots showing the effect of salt concentration on the performance of 80 finger millet genotypes. **(A)** Variation in grain yield (kg ha^-^¹) under control (0 dS m^-^¹), moderate salinity (6 dS m^-^¹), and high salinity (10 dS m^-^¹) and **(B)** variation in fodder yield (kg ha^-^¹) under the same treatments. Statistical differences among treatments were assessed using Kruskal–Wallis test.

**Figure 1** visually shows the significant impact of the imposed salinity gradient on both grain and dried fodder yield, consistent with the ANOVA results reported in **Table 1**. The boxplots for GYLD (A) and DFYLD (B) show a distinct, negative pattern between increasing saline water concentration and overall yield. For GYLD, the average yield decreased sequentially across the treatments, with Kruskal–Wallis test confirming highly significant differences between all three pairs of treatments (*p* < 0.05 for all comparisons). The most substantial shift was observed between the mild stress (6 dS/m) and high salt stress (10 dS/m) environments, where the entire yield distribution shifted to a much lower range, interpreting severe-stress-induced reduction of grain production across the entire germplasm. The pattern for DFYLD (B) followed a similar trend, showing significant differences between all treatments (*p* < 0.05). However, while the mean DFYLD decreased with increasing salinity, the proportional drop between the zero stress (control) and high salt stress (10 dS/m) environment was visually less severe than that observed for GYLD. This results implies that the physiological impact of salinity stress primarily targeted reproductive allocation and grain filling, with less severe inhibition of overall vegetative biomass accumulation.

### Traits contribution, correlation, and identification of potential stress-tolerant finger millet genotypes

**Figure 2:**
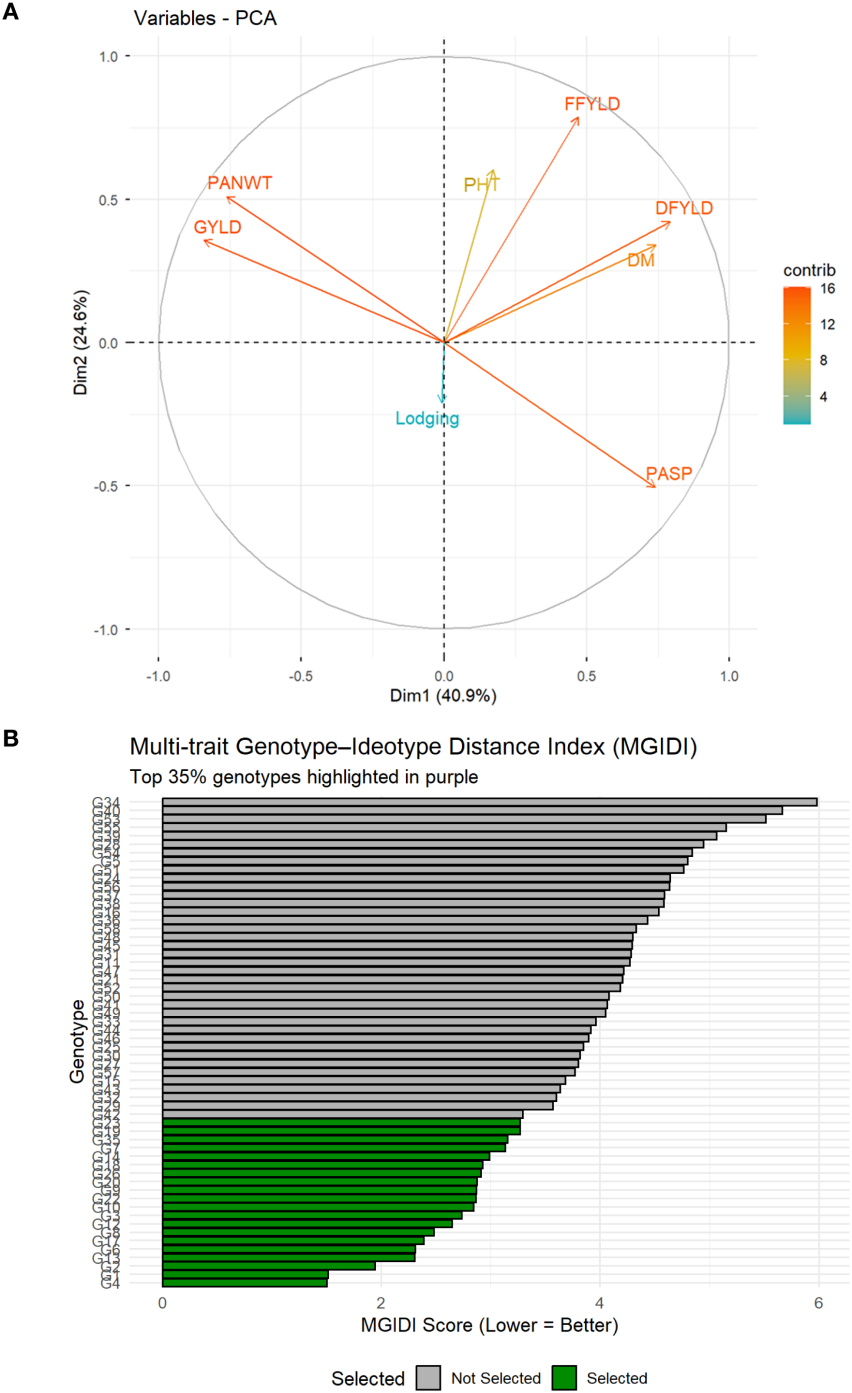
Multivariate analysis of finger millet genotypes and ideotype-based selection. **(A)** Trait correlations and their contributions derived from principal component analysis and **(B)** bar plots of selected salt-tolerant genotypes at 35% selection intensity. DM, days to maturity; DFYLD, dry fodder yield; FFYLD, fresh fodder yield; GYLD, grain yield; NDVI, normalized difference vegetation index; PASP, plant aspect; PHT, plant height; SC, stand count.

The mean performance of the 80 finger millet genotypes for GYLD under the three saline water treatments, alongside the calculated stress tolerance index (STI), which served as the first selection criterion, is presented in **Supplementary Figure S1**. Genotypes exhibited a wide range of responses to saline water stress, confirming the highly significant G × T interaction effect observed in the ANOVA model (**Table 2**). The STI values ranged drastically from a positive index in 58 genotypes to a negative index in 22 genotypes truncated to zero, justifying the index’s utility in discriminating between tolerant and susceptible accessions. Of the 58 genotypes with positive STI, IE 3392 had the highest index (0.92), while IE 2042, IE 4545, and IE 6514 had the least index (0.01). Through principal component analysis (PCA), six traits (PASP, DM, FFYLD, DFYLD, PWT, and GYLD) showed moderate to high contribution to total variation under high salt concentration (**Figure 2A**) and used in the computation of the multi-traits genotype–ideotype idex (MGIDI) for the final selection.

These traits, particularly the reproductive yield (PWT and GYLD) and biomass yield (FFYLD and DFYLD) traits, showed interesting patterns. The PCA correlation circle reveals a generally positive correlation between the reproductive yield traits and the biomass yield traits, as indicated by the acute angle separating their vectors (**Figure 2A**). However, the largest source of variation, Dim 1 (40.9%), segregates these two yield components. The biomass traits were strongly aligned with the positive direction of Dim 1, demonstrating that they are the primary drivers of this major dimension. Conversely, the reproductive yield traits were projected toward the negative direction of Dim 1. This partitioning of the two yield component clusters along the first principal axis shows that distinct genetic factors are governing vegetative growth and reproductive partitioning efficiency.

The final selection was composed of 20 elite finger millet genotype as ideal genotypes for salt tolerance (**Figure 2B**). This selected group demonstrated superior performance compared to the initial population across grain yield for both stress and non-stress treatments. The average GYLD across all environments for these 20 genotypes was 2.07 t/ha. Specifically, under severe saline water stress (10 dS/m), this elite group achieved an average GYLD of 0.76 t/ha, exceeding the overall population mean of 0.29 t/ha (**Table 3**), interpreting effective genetic gain for stress productivity. Similarly, the elite genotypes maintained high dry vegetative biomass, averaging 6.65 t/ha under severe saline water stress. The MGIDI successfully identified several outstanding accessions, led by genotype G4 (IE 3392), which showed the highest STI for GYLD (0.92) and superior performance under severe salinity conditions (1.16 t/ha), identifying it as the most stress-tolerant and stable accession. Other strong performers for GYLD include G3 (IE 7508), G1 (IE 3973), G2 (IE 6473), G9 (IE 6337), G6 (IE 4570), G7 (IE 4646), and G10 (IE 1055) with STI greater than 0.50. For DFYLD, the top performer was G26 (IE 2312-Chk2) with 8.53 t/ha under severe saline water stress. Others include G13 (IE 5870), G35 (IE 518), G8 (IE 4028), G20 (IE 5091), G17 (IE 4671), G12 (IE 4073), and G9 (IE 6337) with STI values greater than 0.6 (**Table 3**).

**Table 3:** Mean performance and stress tolerance index of the best 20 finger millet genotypes for grain and dry fodder yield of the selected finger millet genotypes using MGIDI under zero-, mild-, and high-salinity conditions.

Geno code	Genotype name	Grain yield (t/ha)			STI	Dry fodder yield (t/ha)			STI
		10 dS/m	6 dS/m	0 dS/m		10 dS/m	6 dS/m	0 dS/m	
G23	IE 3104-Chk1	0.44	2.90	3.43	0.31	7.08	9.10	9.79	0.59
G18	IE 501	0.61	2.68	3.34	0.42	5.90	8.25	9.60	0.48
G26	IE 2312-Chk2	0.40	2.06	1.54	0.13	8.53	10.68	12.29	0.90
G4	IE 3392	1.17	3.36	3.81	0.92	6.51	8.78	9.90	0.55
G10	IE 1055	0.79	2.57	2.87	0.47	6.24	8.53	9.83	0.52
G12	IE 4073	0.74	1.97	2.24	0.34	6.90	9.29	10.53	0.62
G1	IE 3973	1.36	3.23	2.59	0.74	6.11	8.22	9.52	0.50
G8	IE 4028	0.83	2.63	2.43	0.42	7.24	9.42	10.75	0.67
G7	IE 4646	0.85	2.44	2.95	0.52	6.66	9.19	10.42	0.59
G17	IE 4671	0.61	2.66	2.44	0.31	7.09	10.13	10.83	0.66
G6	IE 4570	0.90	3.30	2.87	0.54	5.72	7.87	9.15	0.45
G19	IE 4797	0.58	2.69	2.86	0.34	6.54	8.93	10.69	0.60
G3	IE 7508	1.25	3.07	3.37	0.87	5.29	7.49	8.97	0.41
G20	IE 5091	0.56	2.63	3.56	0.41	7.37	9.66	10.55	0.67
G22	IE 2457	0.49	2.62	3.25	0.33	6.73	8.61	9.88	0.57
G35	IE 518	0.32	2.13	2.30	0.15	7.20	9.52	11.70	0.72
G14	IE 7320	0.71	3.14	2.06	0.30	5.62	7.91	9.06	0.44
G13	IE 5870	0.72	2.29	2.50	0.37	8.05	9.66	11.11	0.77
G9	IE 6337	0.81	1.86	3.27	0.55	6.86	8.98	10.46	0.61
G2	IE 6473	1.26	3.15	2.49	0.65	5.38	7.71	8.67	0.40
Mean	Selected	0.77	2.67	2.81	0.45	6.65	8.90	10.18	0.59
Mean	Population	0.29	1.88	2.19		6.98	9.55	10.81	

0 dS/m, zero salinity level; 6 dS/m, mild salinity level; 10 dS/m, high salinity level; STI, stress tolerance index; 0 dS/m, zero stress; 6 dS/m, mild salt stress; 10 dS/m, high salt stress.

### Analysis of variance and mean performance from preliminary trial evaluation

**Table 4** presents the mean squares from the combined ANOVA performed on the 20 elite finger millet genotypes, which serves to validate the success of the multi-trait selection approach by confirming the remaining genetic variability and stability within this superior subset. The ANOVA confirmed that the genotype mean squares were highly significant (*p* < 0.001 or 0.01) for measured traits, including GYLD, DFYLD, FFYLD, PHT, DM, PASP, and NVDI. Similarly, the treatment (0 dS/m, 6 dS/m, 10 dS/m, and 50% irrigation) mean square was highly significant for all traits, implying that even the most resilient genotypes still exhibit measurable phenotypic responses to the imposed stress gradient. The genotype-by-treatment (G × T) interaction mean square was non-significant for the primary yield traits, GYLD and DFYLD, as well as for DM, PHT, NDVI, and FFYLD. This lack of significant interaction validates the successfully identified subset of accessions with stable performance across the saline water levels, effectively minimizing the rank changes that complicate breeding. The G × T interaction was only highly significant for PASP. The success of the selection process is further highlighted by the increase in heritability (*H*^2^) estimates within the elite subset of finger millet. The heritability estimates were high, ranging from 0.78 for GYLD to 0.97 for PHT, which represents a substantial gain in genetic control compared to the initial population estimates (e.g., GYLD increased from 0.60 to 0.78). This suggests that selection effectively purified the genetic background of the desired traits, significantly increasing the predictability and reliability of selection in future cycles. The experimental precision for the validation trial was acceptable, with the coefficient of variation (CV) being moderate, particularly for the biomass yields (DFYLD = 26.40%, FFYLD = 24.14%), which is typical for highly variable yield components.

**Table 4:** Mean square from the combined analysis of variance grain yield and other agronomic traits assessed for 20 elite finger millet genotypes.

Source of variation	DF	SC	DM	PASP	PHT	NVDI	FFYLD	DFYLD	GYLD
Genotype	19	31.94	44.56***	3.87***	628.13**	332.90***	353.35***	0.33***	4.54***
Treatment	3	579.99***	582.57***	9.17***	304.49**	913.57***	75.46	0.91***	78.99***
Genotype × treatment	57	16.88	7.84	0.82***	86.34	71.18	65.55	0.07	0.97
Residual variance		21.70	4.89	0.46	85.78	0.00	45.28	4,705.43	0.98
CV		7.76	1.65	16.31	9.09	7.30	24.14	26.40	19.19
Grand mean		60.00	134.13	4.14	101.86	0.67	27,871	8,217	5,154
Heritability		0.32	0.82	0.79	0.97	0.82	0.82	0.79	0.78

SC, stand count; DM, days to maturity; PASP, plant aspect; PHT, plant height; NVDI, normalized vegetative differential index; FFYLD, fresh fodder yield; DFYLD, dry fodder yield; GYLD, grain yield; ns, not significant (*P* > 0.05); **, significance at probability of 0.01; ***, significance at probability of 001.

### Performance of the elite finger millet genotypes for reproductive and biomass yields under high saline water and managed water-deficit conditions

**Figure 3:**
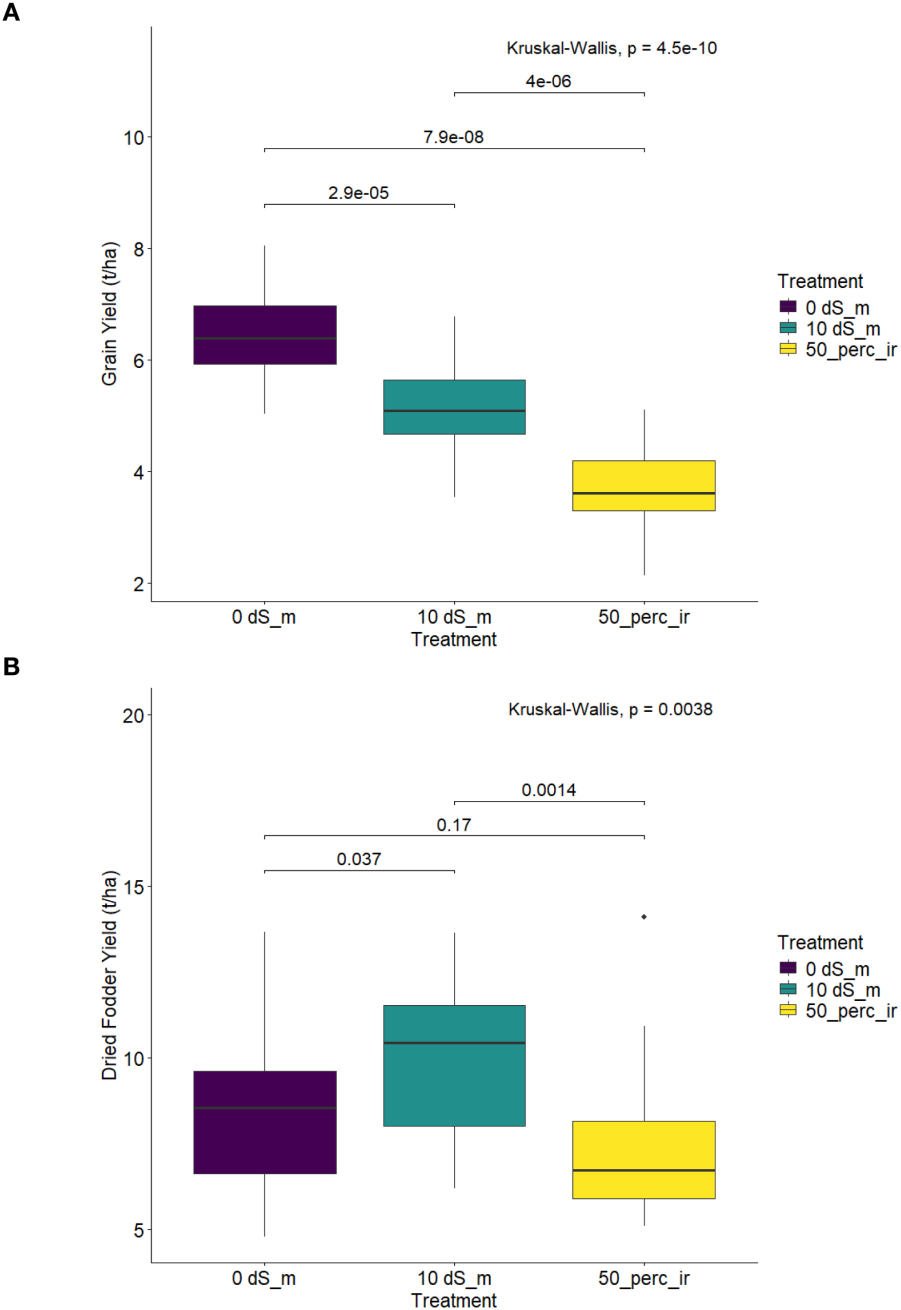
Box plots showing the performance of 20 elite finger millet genotypes under stress conditions. **(A)** Grain yield under optimum (0 dS m^-^¹), severe salinity (10 dS m^-^¹), and induced drought (50% irrigation) environments and **(B)** dry fodder yield under the same conditions. Statistical differences were assessed using Kruskal–Wallis test.

**Figures 3A, B** visually represent the average performance of the selected 20 elite finger millet for GYLD and DFYLD under optimum and stress environments including zero stress conditions (0 dS/m), severe salinity (10 dS/m), and induced drought stress (50% irrigation). For GYLD (**Figure 3A**), Kruskal–Wallis test showed highly significant differences among all three environments. The yield penalty was most severe under induced drought stress (50% irrigation), where the mean GYLD was significantly lower than the yield under severe salinity (*p* < 0.001). This finding implies that for the elite subset, the water deficit imposed by 50% irrigation was the most limiting factor for grain production, resulting in a yield penalty exceeding that imposed by the high osmotic stress of 10 dS/m salinity. The pattern for DFYLD (**Figure 3B**) also showed significant overall treatment effects (*p* < 0.001). However, the vegetative component displayed greater stability between the two stress types. While DFYLD was significantly reduced under stress compared with the control treatment, the mean yield under severe water salinity of 10 dS/m was not significantly different from the yield under drought stress (*p* = 0.17). This non-significant difference between the two stress types suggests that the elite subset possesses a stable, robust physiological mechanism for vegetative biomass maintenance that is equally resilient to the osmotic stress caused by both high salinity and water deficit.

The comparative performance of the elite genotypes quantifying their yield and resilience under control, severe water salinity (10 dS/m), and managed water-deficit stress (50% irrigation), alongside the calculated percentage yield losses, is presented in **Table 5**. Across the elite panel, the average GYLD dropped from 6.48 t/ha (control) to 5.14 t/ha under salinity (10 dS/m), resulting in an average yield loss of 20.4%. The grain yield penalty was even higher under water-deficit stress, where GYLD decreased to 3.69 t/ha, corresponding to an average loss of 42.7%. Similarly, the average DFYLD loss was 14.4% under high saline water treatment and 22.9% under water-deficit stress, confirming that, even in the elite subset, the water-deficit stress imposed a higher overall limitation on both reproductive and vegetative growth compared to salinity.

**Table 5:** Mean performance of selected elite genotypes and yield loss for grain and dry fodder yield under salinity and drought conditions.

	Grain Yield t/ha			Loss (%)	Dry Fodder Yield t/ha			Loss (%)
Genotype	Control	10 dS/m	50_perc_ir	Salinity/Drought	Control	10 dS/m	50_perc_ir	Salinity/Drought
IE 1055	6.64	4.64	4.18	30.11/37.1	7.47	7.42	7.93	0.67/-6.14
IE 2312-Chk2	5.54	4.40	4.20	20.64/24.22	12.75	13.67	14.11	-7.2/-10.66
IE 2457	6.35	4.68	3.04	26.22/52.09	7.68	9.11	6.90	-18.68/10.1
IE 3104-Chk1	5.48	5.00	3.58	8.72/34.66	9.05	4.90	5.89	45.86/35
IE 3392	8.05	6.22	4.38	22.74/45.57	13.63	9.80	5.39	28.14/60.49
IE 3973	7.36	5.01	3.64	31.9/50.56	6.38	9.21	6.49	-44.44/-1.7
IE 4028	7.85	5.23	4.50	33.42/42.69	12.04	9.96	8.15	17.25/32.27
IE 4073	6.40	5.61	2.97	12.34/53.66	11.85	9.25	5.71	21.89/51.8
IE 4570	5.59	4.60	3.96	17.65/29.08	11.43	12.43	10.21	-8.76/10.65
IE 4646	6.03	5.32	4.15	11.9/31.3	8.12	6.64	6.90	18.29/15.1
IE 4671	6.38	4.81	3.33	24.68/47.89	9.50	5.38	5.20	43.43/45.27
IE 4797	5.27	4.06	2.13	23.06/59.57	6.18	4.78	5.10	22.68/17.41
IE 501	7.41	6.77	5.10	8.64/31.25	9.96	7.32	8.94	26.53/10.29
IE 5091	6.29	6.25	3.33	0.77/47.19	10.93	6.55	6.04	40.12/44.78
IE 518	6.84	5.82	4.15	14.96/39.33	9.58	9.55	8.14	0.35/15.06
IE 5870	7.48	5.22	2.57	30.25/65.66	11.22	7.96	6.48	29.06/42.28
IE 6337	6.31	5.76	4.58	8.82/27.51	12.13	6.93	10.93	42.89/9.9
IE 6473	6.84	4.80	3.46	29.91/49.43	11.22	10.85	6.56	3.28/41.54
IE 7320	6.61	5.16	3.18	21.96/51.85	11.01	9.30	5.91	15.59/46.33
IE 7508	5.03	3.54	3.35	29.7/33.52	6.34	5.63	7.06	11.18/-11.46
**Average**	**6.48**	**5.14**	**3.69**	**20.4/42.7**	9.92	8.33	7.40	**14.4/22.9**

The individual genotype performance revealed excellent resilience from some of the elite genotypes—for example, genotype IE 501 demonstrated outstanding stability by yielding higher than the average of the elite genotypes and showing yield losses lower than the average for the group under control, 10 dS/m, and 50% irrigation. Genotypes IE 2312-ChK2 and IE 4570, among others, showed high DFYLD and minimal losses under salinity and water-deficit stresses The genotype IE 6337 demonstrated outstanding stability, exhibiting the lowest GYLD losses under both saline water (8.82%) and water-deficit stress (27.51%). In addition to IE 6337, the genotypes IE 3973, IE 2457, IE 4570, and IE 2312-Chk2 showed compensatory growth responses with gains in DFYLD under salinity (-44.44%, -18.68%, -8.76%, and -7.2%, respectively) and minimal DFYLD loss (< 15%) under water-deficit stress.

### Identification of finger millet genotypes with stable performance across research conditions

**Figure 4:**
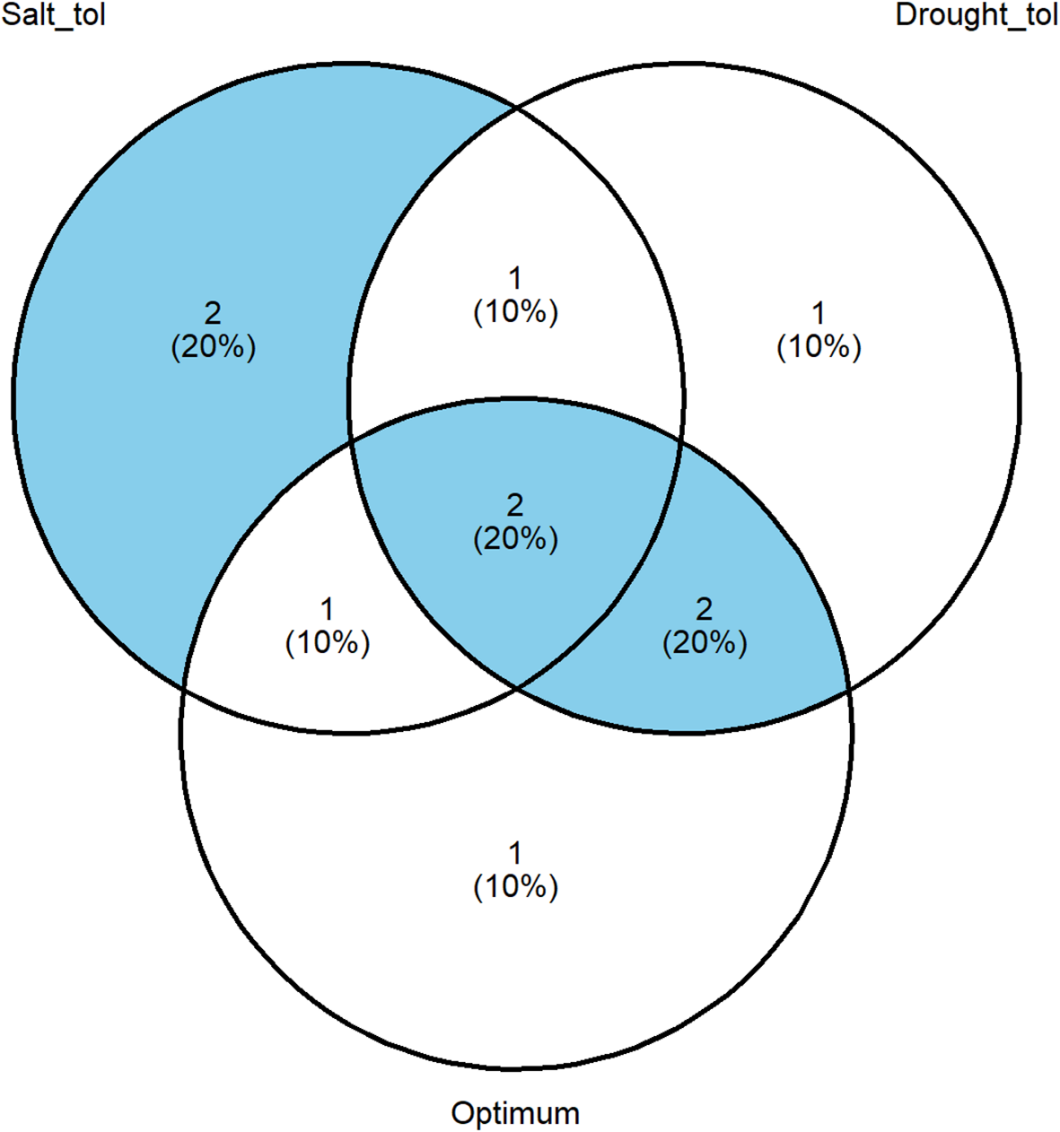
Venn diagram illustrating genotypes/ideotypes with broad adaptation across optimum, high-salinity, and drought stress environments. Genotypes located at the intersections exhibit combined tolerance to multiple stress conditions.

The identification of a stably performing genotype using the multi-trait selection through MGIDI independently across the control, severe salinity (10 dS/m), and drought (50% irrigation) environments is presented in **Supplementary Figure S1**. From each of the research conditions, six finger millet genotypes were selected, representing the top 30% selection bracket. The Venn diagram constructed with the six genotypes from each selection results identified two accession (IE 4570 and IE 4028) at the intersection of the three circles as the most broadly adapted genotypes (**Figure 4**). These genotypes have good ideotype qualities under the three conditions of research (optimum, water salinity, and water-deficit stress). In addition to this two genotypes, several other genotypes were also identified as stable genotypes for other combinations including high salinity and drought stress (IE 6337), drought and optimum treatment (IE 2312-Chk2 and IE 518), and high salinity and optimum treatment (IE 4073) (**Figure 4**).

### Relationship among measured traits under optimum treatment, high salinity, and water-deficit stress

**Figure 5:**
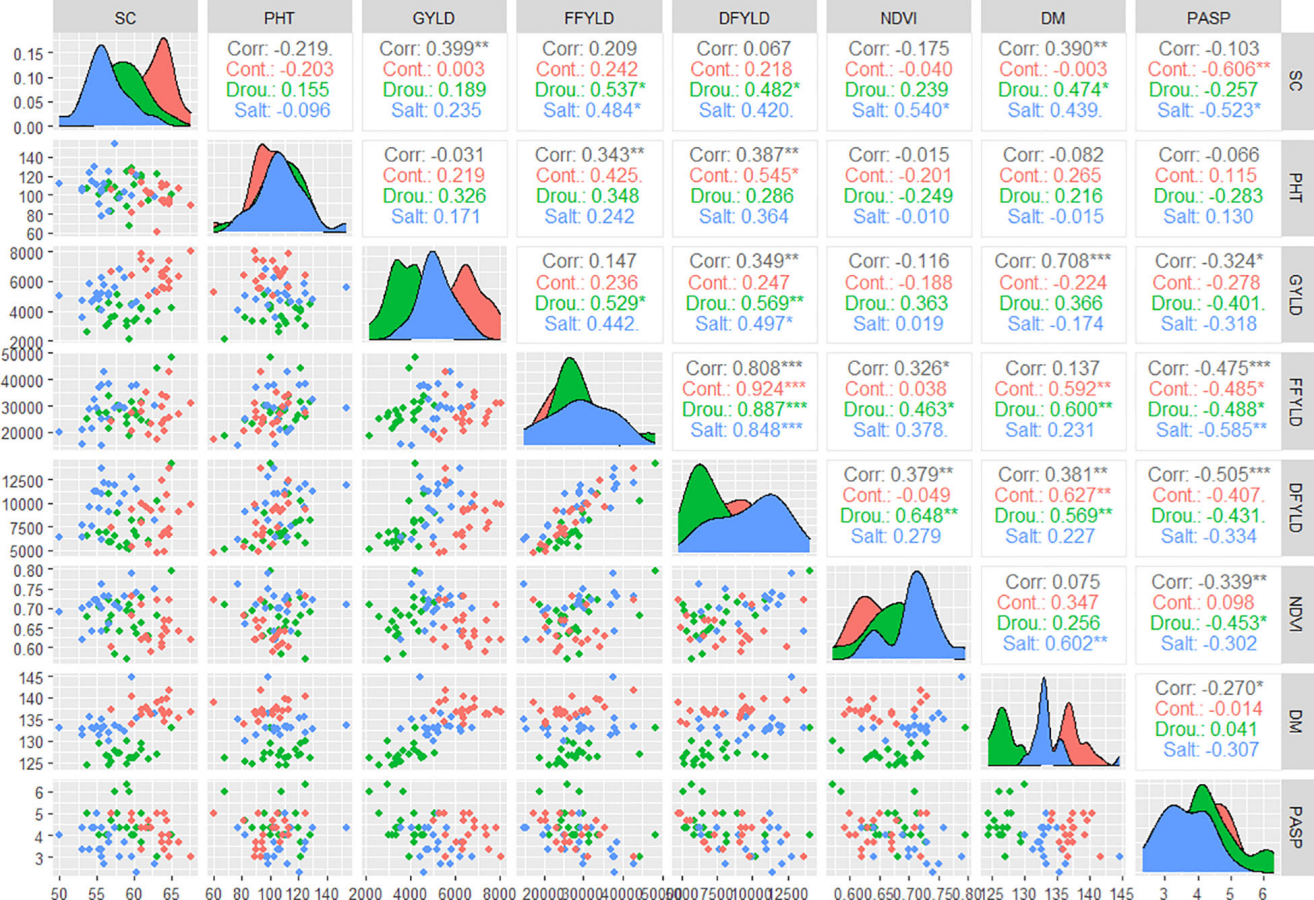
Correlation matrix showing phenotypic relationships among agronomic traits in finger millet under control, salinity, and drought stress environments. DM, days to maturity; DFYLD, dry fodder yield; FFYLD, fresh fodder yield; GYLD, grain yield; NDVI, normalized difference vegetation index; PASP, plant aspect; PHT, plant height; SC, stand count. *, **, and *** indicate significance at *P* ≤ 0.05, 0.01, and 0.001, respectively.

The correlation matrix in **Figure 5** revealed strong and significant relationships among several traits measured under control (optimum), water-deficit, and saline water conditions. Grain yield showed a positive and significant association with SC (*r* = 0.399**), DFYLD (*r* = 0.349**), and DM (*r* = 0.708**) across treatments, suggesting that plots with higher population density, late maturing plants and those with higher dry fodder yield tended to produce higher grain yields. Under drought stress, GYLD was positively correlated with FFYLD (*r* = 0.529*) and DFYLD (*r* = 0.569*), implying that genotypes maintaining biomass production under stress also sustained a higher grain yield. Similarly, under salinity stress, GYLD maintained a positive and significant correlation with DFYLD (*r* = 0.497*), highlighting a consistent relationship between yield and fodder-related traits across stress conditions. A strong positive correlation was also observed between FFYLD and DFYLD across environments (*r* = 0.808***), reflecting their close physiological relationship. In contrast, PASP exhibited a negative and significant correlation with GYLD (*r* = –0.324*) and with biomass yields (*r* = –0.475*** and –0.505*** for FFYLD and DFYLD, respectively), suggesting that plants with a more desirable appearance (lower PASP scores) tended to perform better for yield and biomass (**Figure 5**).

### Genotype-by-traits relationship

**Figure 6:**
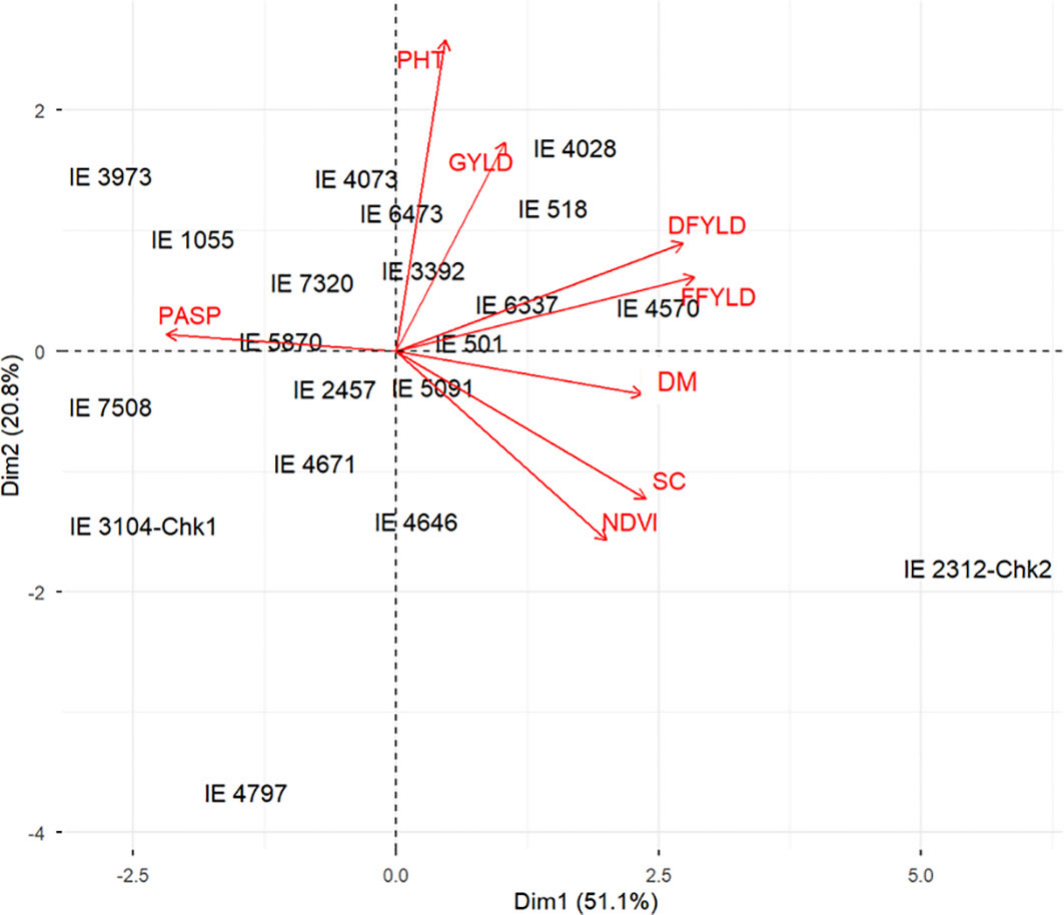
Genotype-by-trait (GT) biplot of 20 elite finger millet genotypes based on principal component analysis, illustrating relationships among genotypes and measured agronomic traits. DM, days to maturity; DFYLD, dry fodder yield; FFYLD, fresh fodder yield; GYLD, grain yield; NDVI, normalized difference vegetation index; PASP, plant aspect; PHT, plant height; SC, stand count.

The genotype-by-trait biplot, derived from the principal component analysis (PCA) performed exclusively on the 20 elite finger millet accessions, visually summarizes the remaining genetic structure and performance relationships within the selected germplasm (**Figure 6**). The first two principal components (Dim 1and Dim 2) captured 71.9% of the total genetic variance. The relationships among the grain yield and biomass traits remain highly consistent. A strong positive correlation is affirmed by the acute angles separating the vectors for GYLD, DFYLD, FFYLD, and PHT. This implies that the genetic factors governing high biomass and high grain yield are tightly linked within the elite subset. The vector for PASP is positioned nearly opposite the GYLD vector, confirming a fundamental negative correlation between this morphological trait and final grain yield. The positioning of the finger millet genotypes within this elite space identifies key superior accessions. The genotypes IE 4028, IE 518, and IE 6337 are situated in the high-performance quadrant, strongly aligned with the favorable yield and biomass vectors, also affirming their high multi-trait productivity. Notably, the check genotype IE 2312-Chk2 remains an outlier, positioned far to the right along Dim 1, primarily associated with SC and NDVI, a measure of plant vigor and greenness. This visual evidence confirms that while the MGIDI selection successfully minimized detrimental genotype-by-treatment interaction (**Table 4**), exploitable genetic variability for key traits persists among the selected 20 elite genotypes.

## Discussion

The development of climate-resilient crops for hyper-arid environments requires a shift from evaluating single traits to a holistic assessment of genotype performance under multiple stressors. In this study, we integrated stress tolerance parameters and multi-trait selection indices (MGIDI) to identify finger millet genotypes capable of maintaining productivity under extreme salinity and water-deficit conditions. Our findings revealed significant genotype-by-treatment interactions and a distinct decoupling of vegetative and reproductive resilience, providing a framework for breeding dual-purpose cereals in marginal lands.

Current research in plant stress biology highlights that surviving harsh environments is not the result of a single trait, but rather a web of interconnected physiological and molecular responses. When salinity and drought strike simultaneously, plants face intense oxidative pressure as reactive oxygen species (ROS) build up. To maintain growth, the plant must deploy an efficient antioxidant system to keep these levels in check (Lin et al., 2024; Mittler et al., 2022). Breeders are now prioritizing these redox signaling pathways; by understanding how a plant perceives and translates environmental cues into metabolic changes, we can better develop crops resilient to a changing climate (Hasanuzzaman et al., 2020, 2021; Waszczak et al., 2018).

### Physiological response and efficacy of salinity stress

Salinity is one of the biggest challenges to finger millet production in dry lands. In the current study, the pronounced reduction in grain yield and panicle weight with increasing salinity confirmed the efficacy of the imposed salt gradient in differentiating genotypic performance. The magnitude of grain yield loss from approximately 2.19 t/ha under control conditions to 0.29 t/ha under 10 dS/m saline water treatment demonstrates the severity of ionic and osmotic constraints imposed by salinity (**Table 1**; **Figure 1A**). However, the relatively smaller proportional reduction in dry forage yield compared to grain yield highlights a crucial physiological imbalance. Under salinity stress, plants preserved vegetative growth but failed to sustain efficient reproductive partitioning (**Table 1**; **Figure 1B**). This decoupling of reproductive and vegetative productivity suggests that salinity in finger millet primarily disrupts assimilate translocation and grain filling rather than photosynthetic biomass accumulation. This disparity strongly suggests that the primary physiological impact of salinity stress on the finger millet germplasm is not a proportional suppression of overall vegetative biomass accumulation but inhibition of reproductive partitioning and grain filling (Munns and Tester, 2008; Parida and Das, 2005). This phenomenon, where vegetative biomass is maintained at the expense of grain yield, is common in crops under osmotic stress (Flowers and Colmer, 2015; Gupta and Gopalakrishna, 2010) and reinforces the need to target traits beyond simple biomass for tolerance selection.

Recent studies further indicate that such reproductive failure under salinity and drought is closely linked to redox imbalance and impaired carbohydrate metabolism in reproductive tissues. Excess ROS accumulation in developing panicles and florets disrupts sucrose transport, starch biosynthesis, and hormonal balance, leading to poor grain set and reduced sink strength (Renzetti et al., 2022; Zandalinas et al., 2020). The maintenance of vegetative growth observed in this study may therefore reflect more robust antioxidant capacity and osmotic protection in source tissues, while reproductive organs remain more sensitive to oxidative and osmotic perturbations.

The slight but significant delay in maturity under severe salinity is consistent with a generalized stress-avoidance mechanism documented in other cereals under osmotic stress (Shavrukov, 2013), in which plants postpone reproductive development to allow more time for osmotic adjustment or tissue repair. Such phenological plasticity, though beneficial for short-term survival, ultimately constrains yield under prolonged or high-intensity stress, a finding previously documented in other cereals under osmotic stress (Zhu, 2016).

At the physiological level, delayed phenology under stress has been associated with altered redox-controlled signaling pathways involving abscisic acid (ABA), sugar signaling, and transcriptional regulators of flowering time. These pathways allow plants to temporarily prioritize survival over reproduction but often at the cost of final yield stability under chronic stress exposure (Foyer et al., 2017; Gupta et al., 2020).

### Genetic variation and implications for multi-trait selection

The significant genotypic variation across all measured traits confirmed that the evaluated finger millet panel harbors broad genetic diversity for both yield and adaptive physiological traits under saline water stress. The strong genotype-by-treatment interaction for key traits including grain and fresh biomass yield, lodging, and flowering further emphasizes the genotype-specific nature of salinity tolerance (**Table 2**). This instability of rank performance across salinity levels indicates that selection under non-stress conditions would not reliably predict performance under saline environments. This finding is consistent with genotype-specific osmotic and ionic exclusion mechanisms observed in other crops (Blum, 2010).

Emerging evidence suggests that such genotype-specific responses are driven not only by differences in ion transport or water relations but also by variability in redox buffering capacity, antioxidant enzyme activity, and the regulation of stress-responsive transcriptional networks. Genotypes capable of sustaining redox homeostasis under fluctuating stress conditions tend to exhibit a more stable performance across environments, reinforcing the value of multi-trait selection approaches that indirectly capture these complex physiological attributes (Hasanuzzaman et al., 2020; Mittler et al., 2022).

Importantly, the moderately high broad-sense heritability (*H*² = 0.60) for grain yield and plant aspect (**Table 2**) suggested that direct phenotypic selection for these traits would be effective, provided that evaluations are conducted under representative salinity levels. This justified their prominent weighting in the multi-trait genotype–ideotype distance index (MGIDI), which integrates correlated traits into a single selection criterion (Acquaah, 2012; Olivoto and Nardino, 2021).

### Selection efficiency and validation of genetic purification

The application of MGIDI successfully identified genotypes combining yield potential and stress resilience. The elite subset of 20 genotypes achieved nearly a threefold yield (0.77 t/ha) compared to the reference population mean under severe water salinity, confirming the effectiveness of multi-trait selection in complex stress environments (**Table 3**). The superior performance of these genotypes is likely underpinned by robust physiological mechanisms, as evidenced by their ability to maintain better plant aspect scores and higher NDVI values compared to the wider population. These traits serve as reliable proxies for chlorophyll retention and overall plant vigor under stress.

Chlorophyll retention and sustained canopy greenness under stress are increasingly recognized as indicators of effective redox regulation and delayed stress-induced senescence. Recent work has shown that genotypes with enhanced antioxidant capacity and efficient ROS-scavenging systems maintain higher photosynthetic competence and delayed leaf senescence under salinity and drought stress, contributing to improved biomass accumulation and yield stability (Hasanuzzaman et al., 2021; Mittler et al., 2022; Zandalinas et al., 2020).

The subsequent validation analysis, showing a non-significant genotype-by-treatment interaction within this elite subset (**Table 4**), indicates that the selected group possesses improved stability and reduced sensitivity to environmental changes. This confirms that the MGIDI successfully minimized the undesirable rank changes that complicated the initial population analysis (Olivoto and Lúcio, 2020). Moreover, the increase in heritability for grain yield from 0.60 in the base population to 0.78 in the elite group signifies a marked purification of the genetic background. This is an expected outcome in breeding when superior alleles controlling additive genetic variance are concentrated through selection. Such improvement in genetic predictability is a fundamental goal of recurrent selection in stress breeding programs (Bernardo, 2002; Falconer and Mackay, 1996).

### Comparative stress response and identification of superior accessions

The comparative analysis of the elite genotypes under salinity as opposed to managed water-deficit stress provided unique insights. When compared across water salinity and managed water-deficit treatments, the elite genotypes displayed contrasting physiological responses. Water-deficit stress imposed a much greater yield penalty (42.7% loss) than water salinity (20.4% loss), underscoring that osmotic limitation rather than ionic toxicity is the predominant yield constraint in the finger millet germplasm (**Table 5**).

This observation aligns with recent multi-stress studies demonstrating that drought-induced oxidative stress often exceeds that caused by moderate salinity due to severe limitations on carbon assimilation and enhanced ROS production under water deficit. Effective osmotic adjustment through compatible solute accumulation (e.g., proline, sugars) and redox buffering is therefore central to sustaining vegetative growth under both stresses (Farooq et al., 2019; Zörb et al., 2019).

However, dry fodder yield remained statistically comparable under both stresses, indicating that the selected genotypes possess strong osmotic adjustment and water-use efficiency mechanisms sustaining vegetative growth despite reduced grain production. This suggests that the selected germplasm possesses excellent shared mechanisms for osmotic adjustment that benefit vegetative growth under both types of stresses (Chaves et al., 2002; Farooq et al., 2009; Munns and Tester, 2008), while grain filling remains disproportionately sensitive to severe water deficit.

The integration of MGIDI with stress tolerance indices enabled the identification of high-performing, stable accessions with broad adaptation across environments. Genotype G4 (IE 3392) emerged as the most promising salt-tolerant line, combining superior yield (1.16 t/ha at 10 dS/m) and a high stress tolerance index (0.92) (**Table 5**). Additionally, IE 4570 and IE 4028 were identified as multi-stress-tolerant genotypes occupying the intersection of optimal, saline, and drought environments in the Venn diagram analysis (**Figure 4**). These accessions provide immediate genetic resources for introgression and pre-breeding efforts aimed at improving salt and drought resilience in finger millet. This group of identified elite accessions provides immediate genetic resources for future finger millet breeding efforts aimed at developing climate-resilient varieties (Golldack et al., 2011; Kumar et al., 2022).

### Trait associations and breeding implications

The consistently strong positive correlation between grain yield and dry fodder yields across all environments suggests a shared genetic basis for overall productivity and stress resilience (**Figure 5**). This trait association is particularly valuable for breeding dual-purpose finger millet types capable of providing both grain and forage under marginal conditions (Reynolds et al., 2022).

From a physiological perspective, such coordinated performance reflects integrated regulation of carbon allocation, osmotic balance, and redox homeostasis across source and sink tissues. Recent breeding-oriented reviews emphasize that selecting for traits indirectly linked to redox stability and osmotic regulation can substantially enhance genetic gain under combined stress environments (Lin et al., 2024; Mittler et al., 2022).

The genotype-by-treatment biplot further confirmed this pattern, showing that high-yielding accessions tended to cluster with high-biomass types under both saline and drought conditions (**Figure 6**). Collectively, these findings demonstrate that integrating multi-trait selection indices with stress tolerance parameters is a powerful approach to enhance selection efficiency and genetic gain under complex abiotic stresses. The identified elite genotypes form a valuable foundation for developing climate-resilient finger millet cultivars suitable for production in saline-prone and water-limited agro-ecologies. By moving beyond a simple germplasm screening to a targeted validation of an elite subset, this study provides a concrete rather than speculative framework for identifying genotypes where both reproductive and vegetative resilience are successfully balanced. The stability of these lines across multiple stress regimes confirmed their potential as high-impact candidate parental lines for the next generation of climate-smart cereals.

## Conclusions

This study successfully demonstrated the high impact of the imposed salinity gradient on the finger millet germplasm and, critically, validated a methodology for selecting accessions with broad-spectrum tolerance to combined salinity and drought stress. The initial evaluation confirmed that the high salinity stress (10 dS/m) drastically reduced the average grain yield by 87% across the population, with the genotype-by-treatment interaction being highly significant for grain yield. The application of the multi-trait genotype–ideotype distance index, informed by the stress tolerance index, effectively filtered the initial germplasm, resulting in a set of 20 elite genotypes that showed a substantial genetic gain, exceeding the population mean under stress by over 167%. The validation trial confirmed the stability of this elite subset, as the genotype-by-treatment interaction was non-significant for grain and fodder yields across stress and optimal environments (optimum, two salinity levels, and drought). Furthermore, the study revealed that for the elite genotypes, drought stress was the single most limiting factor for grain yield, causing a significantly greater average yield loss (42.7%) than 10 dS/m salinity (20.4%). However, the dry vegetative biomass proved equally resilient to both salinity and drought, validating the successful selection of dual-purpose accessions. The Venn diagram analysis ultimately identified a core group of stable accessions, including IE 4028 and IE 4570, which demonstrated a consistently high performance across optimum, salinity, and drought stress, making them ideal genetic resources for breeding programs targeting future climate-resilient finger millet cultivars.

## Data Availability

The raw data supporting the conclusions of this article will be made available by the authors, without undue reservation.

## Supplementary material

Supplementary Figure 1 Bar plots showing the selection of ideal finger millet genotypes using the multi-trait genotype–ideotype distance index (MGIDI). Selections **(A)** under optimum conditions, **(B)** under high salinity stress, and **(C)** under induced drought stress.
