# Correction: Differential response of finger millet accessions to contrasting saline water levels and irrigation regimes under desert conditions

**DOI:** 10.3389/fpls.2026.1839878

**Published:** 2026-06-01

**Authors:** Abidemi Talabi, Nhamo Nhamo, Sumitha Thushar, Prashant Vikram, Hifzurrahman Rahman, Mohammed Shahid, Neeru Sood, Malavika Sudheer, Dheeraj Thikkamaneni, Amna Almarri, Fatma Alsaffar, Deep Galani, Moyeez Alam, Sonia Goel, Rakesh KSingh

**Affiliations:** 1 International Center for Biosaline Agriculture, Dubai, United Arab Emirates; 2 Innovation Oasis, Silal Food and Technology LLC., Abu Dhabi, United Arab Emirates; 3 ICAR - Indian Agricultural Research Institute, New Delhi, India; 4 Department of Biotechnology, Birla Institute of Technology & Science Pilani, Dubai, United Arab Emirates; 5 Zayed University, Dubai, United Arab Emirates; 6 Junagadh Agricultural University, Junagadh, Gujarat, India; 7 Olobion Omics Labs, Abu Dhabi, United Arab Emirates

**Keywords:** arid and semi-arid regions, drought stress, genetic stability, marginal environments, multi-traits genotype-ideotype distance index, salinity stress, stress tolerance index, water scarcity

Affiliation 4 “Department of Biotechnology, Birla Institute of Technology & Science Pilani, Dubai, United Arab Emirates” was erroneously given as “Birla Institute of Technology and Science, Dubai, United Arab Emirates”.

The original version of this article has been updated.

There was a mistake in **Table 5** as published. Unit of Grain Yield and Dry Fodder Yield were quoted in kg/ha. Similarly, the SI units of water salinity were quoted as ds/m. The corrected **Table 5** appears below.

**Table 5:** Mean performance of selected elite genotypes and yield loss for grain and dry fodder yield under salinity and drought conditions.

	Grain Yield t/ha			Loss (%)	Dry Fodder Yield t/ha			Loss (%)
Genotype	Control	10 dS/m	50_perc_ir	Salinity/Drought	Control	10 dS/m	50_perc_ir	Salinity/Drought
IE 1055	6.64	4.64	4.18	30.11/37.1	7.47	7.42	7.93	0.67/-6.14
IE 2312-Chk2	5.54	4.40	4.20	20.64/24.22	12.75	13.67	14.11	-7.2/-10.66
IE 2457	6.35	4.68	3.04	26.22/52.09	7.68	9.11	6.90	-18.68/10.1
IE 3104-Chk1	5.48	5.00	3.58	8.72/34.66	9.05	4.90	5.89	45.86/35
IE 3392	8.05	6.22	4.38	22.74/45.57	13.63	9.80	5.39	28.14/60.49
IE 3973	7.36	5.01	3.64	31.9/50.56	6.38	9.21	6.49	-44.44/-1.7
IE 4028	7.85	5.23	4.50	33.42/42.69	12.04	9.96	8.15	17.25/32.27
IE 4073	6.40	5.61	2.97	12.34/53.66	11.85	9.25	5.71	21.89/51.8
IE 4570	5.59	4.60	3.96	17.65/29.08	11.43	12.43	10.21	-8.76/10.65
IE 4646	6.03	5.32	4.15	11.9/31.3	8.12	6.64	6.90	18.29/15.1
IE 4671	6.38	4.81	3.33	24.68/47.89	9.50	5.38	5.20	43.43/45.27
IE 4797	5.27	4.06	2.13	23.06/59.57	6.18	4.78	5.10	22.68/17.41
IE 501	7.41	6.77	5.10	8.64/31.25	9.96	7.32	8.94	26.53/10.29
IE 5091	6.29	6.25	3.33	0.77/47.19	10.93	6.55	6.04	40.12/44.78
IE 518	6.84	5.82	4.15	14.96/39.33	9.58	9.55	8.14	0.35/15.06
IE 5870	7.48	5.22	2.57	30.25/65.66	11.22	7.96	6.48	29.06/42.28
IE 6337	6.31	5.76	4.58	8.82/27.51	12.13	6.93	10.93	42.89/9.9
IE 6473	6.84	4.80	3.46	29.91/49.43	11.22	10.85	6.56	3.28/41.54
IE 7320	6.61	5.16	3.18	21.96/51.85	11.01	9.30	5.91	15.59/46.33
IE 7508	5.03	3.54	3.35	29.7/33.52	6.34	5.63	7.06	11.18/-11.46
**Average**	**6.48**	**5.14**	**3.69**	**20.4/42.7**	9.92	8.33	7.40	**14.4/22.9**

The original version of this article has been updated.

