# Supplementary figures and images for: Differential response of finger millet accessions to contrasting saline water levels and irrigation regimes under desert conditions
Source: Front Plant Sci. 2026 Feb 27;17:1754820. doi: 10.3389/fpls.2026.1754820 (PMC12983532; doi:10.3389/fpls.2026.1754820)

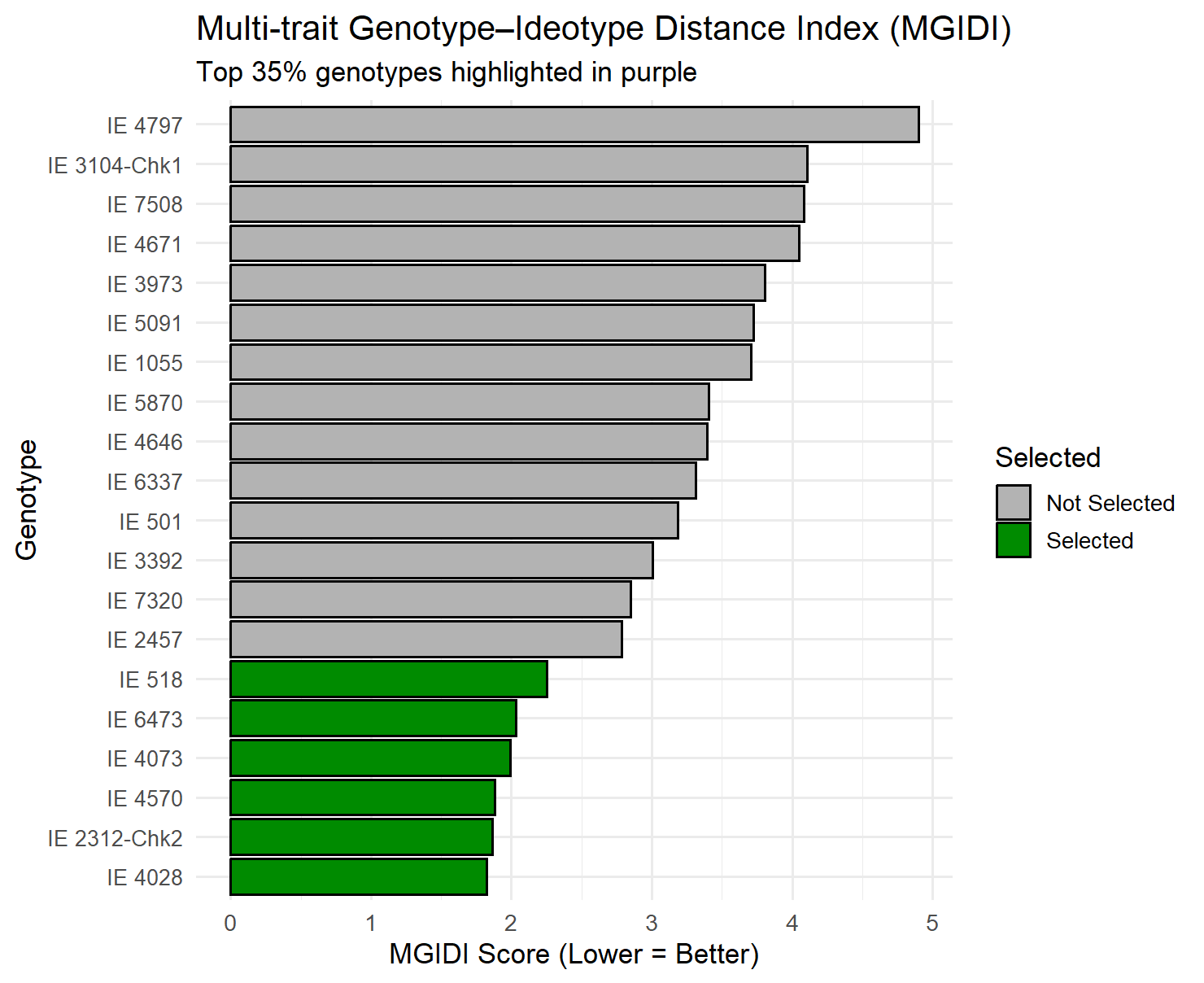

Supplement: Supplementary Figure 1 — Bar plots showing the selection of ideal finger millet genotypes using the multi-trait genotype–ideotype distance index (MGIDI). Selections (A) under optimum conditions, (B) under high salinity stress, and (C) under induced drought stress. [file Image1.tiff]

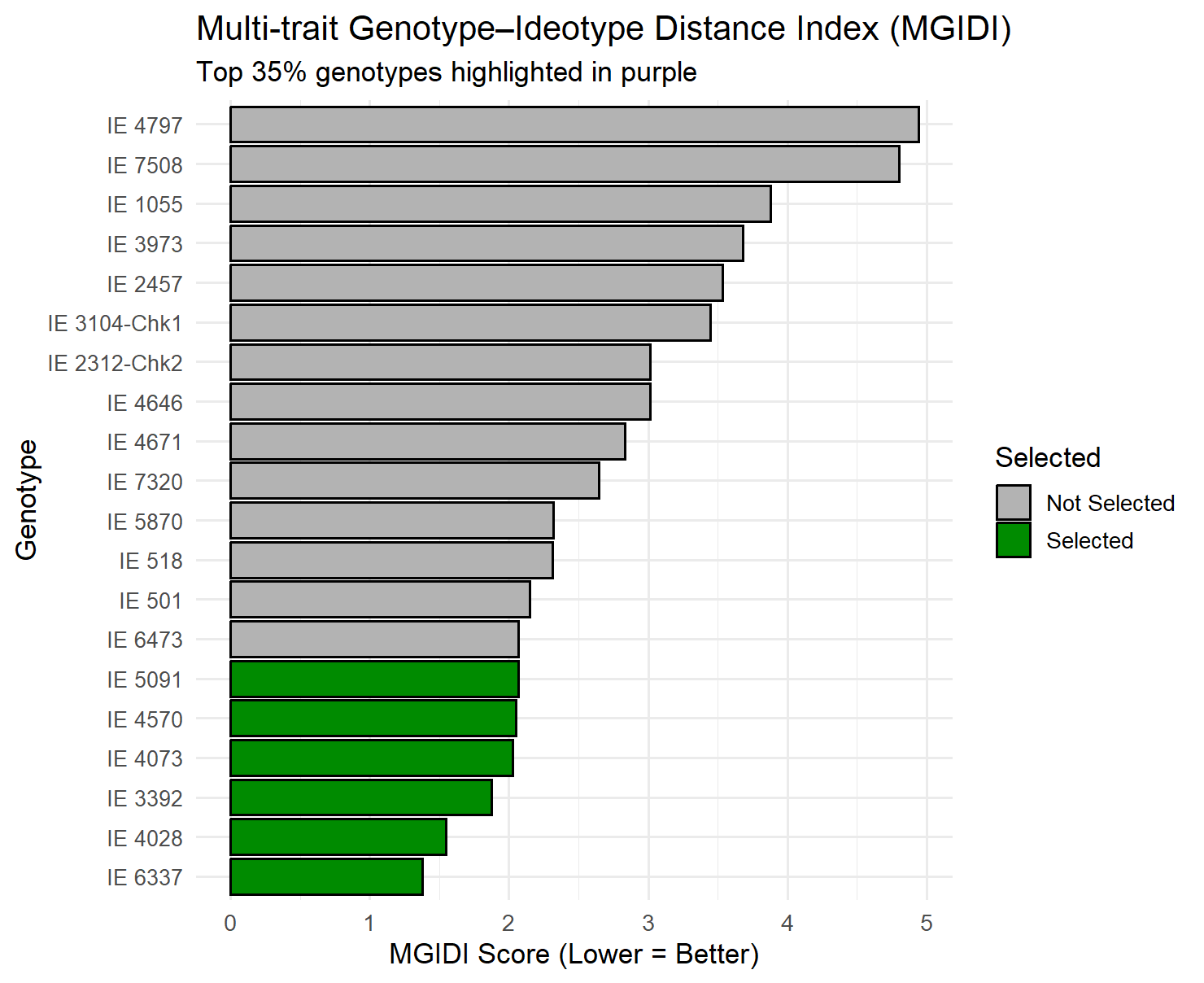

Supplement: Supplementary file 2 [file Image2.tiff]

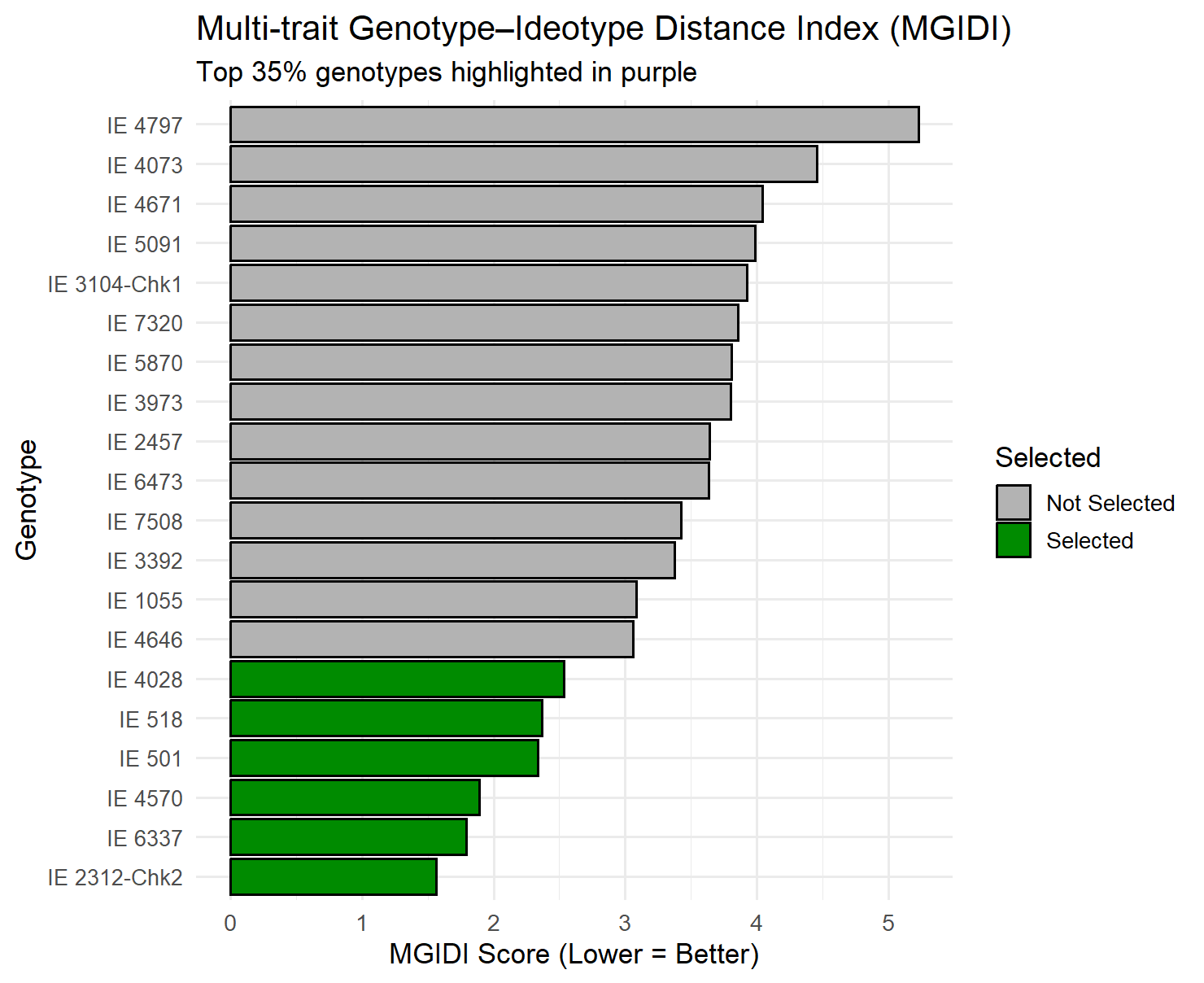

Supplement: Supplementary file 3 [file Image3.tiff]
