# Supplementary material for: Differential response of finger millet accessions to contrasting saline water levels and irrigation regimes under desert conditions
Source: Front Plant Sci. 2026 Feb 27;17:1754820. doi: 10.3389/fpls.2026.1754820 (PMC12983532; doi:10.3389/fpls.2026.1754820)
Supplement: Supplementary file 4 [file DataSheet1.pdf]

Supplementary Table 1. Climatic information of study location during the period 2021-2022.

Months	Range	2021			2022		
		Temp (ºC)	Rainfall (mm)	RH (%)	Temp (ºC)	Rainfall (mm)	RH (%)
January	Mean	18.2	0.9	55.13	19.1	16.7	64.3
	Max	24.5	N/A	78.46	23.7	N/A	84.4
	Min	13.0	N/A	30.46	14.8	N/A	42.1
February	Mean	20	0.3	60.7	20.1	0.9	57.5
	Max	27.1	N/A	84.73	26.0	N/A	81.9
	Min	15.9	N/A	34.02	14.6	N/A	33.0
March	Mean	23.4	0.0	44.39	24.8	0.0	47.4
	Max	32.5	N/A	70.99	31.6	N/A	72.8
	Min	19	N/A	21.43	18.6	N/A	23.4
April	Mean	27.9	5.6	38.4	29.2	2.0	38.8
	Max	36.0	N/A	63.62	36.6	N/A	64.5
	Min	22.2	N/A	17.2	22.4	N/A	16.7
May	Mean	32.3	5.0	41.01	31.3	0.0	39.5
	Max	39.8	N/A	68.22	38.4	N/A	65.5
	Min	25.7	N/A	17.63	24.7	N/A	18.0
June	Mean	34.5	3.1	45.17	34.8	1.7	43.5
	Max	42	N/A	72.08	41.8	N/A	67.7
	Min	28.8	N/A	21.24	28.6	N/A	21.4
July	Mean	35.8	4.3	47.08	34.5	14.8	55.2
	Max	42	N/A	68.14	39.6	N/A	74.5
	Min	31.1	N/A	27.58	30.4	N/A	35.8
August	Mean	35.7	3.3	47.78	35.7	4.1	44.3
	Max	42.1	N/A	72.56	42.0	N/A	67.9
	Min	30.3	N/A	24.63	30.2	N/A	22.3
September	Mean	33.3	4.8	50.42	33.1	4.4	51.9
	Max	40.2	N/A	75.12	39.5	N/A	76.3
	Min	28.5	N/A	25.52	27.7	N/A	27.0
October	Mean	29.6	1.5	53.49	29.7	2.3	51.4
	Max	36.1	N/A	78.49	36.5	N/A	79.4
	Min	24.8	N/A	28.54	23.6	N/A	23.3
November	Mean	24.6	4.7	56.22	26.4	3.3	56.1
	Max	31.0	N/A	79.48	32.2	N/A	79.3
	Min	20.1	N/A	31.9	21.3	N/A	31.9
December	Mean	20.1	6.2	59.26	21.8	6.0	62.0
	Max	26.8	N/A	79.72	26.7	N/A	82.3
	Min	16.6	N/A	36.37	17.2	N/A	40.0

Source: UAE.Stat Data Explorer • Climate - UAE Rainfall, Temperature, and Relative Humidity Indicators

Classification: Public

Supplementary Table 2. Chemical and particle size analysis for soil samples collected from the top 0-30 cm and the subsoil layers 30-60 cm at ICBA research farm in Dubai.

Depth	Clay (%)	Sand (%)	Silt (%)	Organic Matter (%)	pH
0-30 cm	0.73	98.38	0.89	0.55	7.66
30-60 cm	0.43	98.73	0.84	0.28	7.73

Classification: Public

Supplementary Table 3. Comprehensive chemical characterization of irrigation water used in ICBA field experiments (Dubai, UAE), 2024.

Parameter	Unit	Fresh Water	Moderately Saline Water	Moderately (6 dS/m)*	High (10 dS/m)*	Highly Saline Water
General Properties						
pH	–	7.6	7.4	7.39	7.35	7.3
Electrical Conductivity (ECw)	dS m^{-1}	0.6	5	6	10	15
Total Dissolved Solids (TDS)	mg L^{-1}	380	3,200	3840	6400	9,600
Major Cations						
Calcium (Ca^{2+})	meq L^{-1}	2.5	15	17.5	27.5	40
Magnesium (Mg^{2+})	meq L^{-1}	1.8	10	12	20	30
Sodium (Na^{+})	meq L^{-1}	2	35	42.5	72.5	110
Potassium (K^{+})	meq L^{-1}	0.1	1.5	1.6	2	2.5
Major Anions						
Chloride (Cl^{-})	meq L^{-1}	3	40	48	80	120
Sulfate (SO_4^{2-})	meq L^{-1}	2	30	33.5	47.5	65
Bicarbonate (HCO_3^{-})	meq L^{-1}	2.5	4	4.1	4.5	5
Carbonate (CO_3^{2-})	meq L^{-1}	0	0.1	0.11	0.15	0.2
Nitrate (NO_3^{-})	mg L^{-1}	5	10	10.5	12.5	15
Derived Indices						
Sodium Adsorption Ratio (SAR)	–	1.8	8.5	9.17	11.85	15.2
Residual Sodium Carbonate (RSC)	meq L^{-1}	0	0	0	0	0
Adjusted SAR	–	2	9.2	9.88	12.6	16
Trace Elements						
Boron (B)	mg L^{-1}	0.3	1.5	1.65	2.25	3
Iron (Fe)	mg L^{-1}	0.05	0.1	0.105	0.125	0.15
Manganese (Mn)	mg L^{-1}	0.02	0.05	0.053	0.065	0.08
Zinc (Zn)	mg L^{-1}	0.01	0.02	0.021	0.025	0.03

* - derived ionic composition of water at 6 dS/m and 10 dS/m based on known compositions at 0.6 dS/m, 5 dS/m and 15 dS/m.

Classification: Public
